# *Panax notoginseng* alleviates oxidative stress through miRNA regulations based on systems biology approach

**DOI:** 10.1186/s13020-023-00768-y

**Published:** 2023-06-20

**Authors:** Yun Tang, Yi-Gang Chen, Hsi-Yuan Huang, Shang-Fu Li, Hua-Li Zuo, Ji-Hang Chen, Li-Ping Li, Run-Bo Mao, Yang-Chi-Dung Lin, Hsien-Da Huang

**Affiliations:** 1 School of Medicine, The Chinese University of Hong Kong, Shenzhen, Longgang District, Shenzhen, 518172 Guangdong China; 2 Warshel Institute for Computational Biology, The Chinese University of Hong Kong, Shenzhen, Longgang District, Shenzhen, 518172 Guangdong China

**Keywords:** *Panax notoginseng* (PNS or Sanqi), Anti-oxidant, Metabolic disorder, MicroRNA, Systems biology

## Abstract

**Background:**

Herbal medicine Sanqi (SQ), the dried root or stem of *Panax notoginseng* (PNS), has been reported to have anti-diabetic and anti-obesity effects and is usually administered as a decoction for Chinese medicine. Alternative to utilizing PNS pure compound for treatment, we are motivated to propose an unconventional scheme to investigate the functions of PNS mixture. However, studies providing a detailed overview of the transcriptomics-based signaling network in response to PNS are seldom available.

**Methods:**

To explore the reasoning of PNS in treating metabolic disorders such as insulin resistance, we implemented a systems biology-based approach with RNA sequencing (RNA-seq) and miRNA sequencing data to elucidate key pathways, genes and miRNAs involved.

**Results:**

Functional enrichment analysis revealed PNS up-regulating oxidative stress-related pathways and down-regulating insulin and fatty acid metabolism. Superoxide dismutase 1 (SOD1), peroxiredoxin 1 (PRDX1), heme oxygenase-1 (Hmox1) and glutamate cysteine ligase (GCLc) mRNA and protein levels, as well as related miRNA levels, were measured in PNS treated rat pancreatic β cells (INS-1). PNS treatment up-regulated Hmox1, SOD1 and GCLc expression while down-regulating miR-24-3p and miR-139-5p to suppress oxidative stress. Furthermore, we verified the novel interactions between miR-139-5p and miR-24-3p with GCLc and SOD1.

**Conclusion:**

This work has demonstrated the mechanism of how PNS regulates cellular molecules in metabolic disorders. Therefore, combining omics data with a systems biology strategy could be a practical means to explore the potential function and molecular mechanisms of Chinese herbal medicine in the treatment of metabolic disorders.

**Supplementary Information:**

The online version contains supplementary material available at 10.1186/s13020-023-00768-y.

## Background

Herbal drugs have a typical nature involving multi-component and multichannel, and with the aid of next-generation sequencing (NGS), monitoring herbal drug molecular mechanisms in modern diseases is therefore made possible. In past research, it has shown to be a time-efficient and cost-effective way to authenticate herbal drugs such as Traditional Chinese Medicines (TCMs). During the past 15 years, the high throughput of massively parallel sequencing has become a powerful tool, used in various fields, such as clinical genetics, oncology and microbiology [1, 2]. To discuss disorders with highly complex metabolic mechanism, researchers focused on the transcriptomic changes of the pancreatic β cell through NGS-based analysis [3–5] and TCMs have been found to increase insulin synthesis and secretion as well as reduce β cell apoptosis and oxidative stress. As an alternative medicine, TCMs have been practiced for thousands of years and *Panax notoginseng* (PNS) has its fair share of contributions. Studies have shown that ginsenosides can promote insulin synthesis and secretion [6], inhibit β cell apoptosis [7], and reduce inflammation and oxidative stress damage [8].

PNS, also known as “Sanqi (SQ)” in Chinese, is composed of several compounds including saponins, flavonoids, cyclopeptides, sterols, polyacetylenes, amino acids, volatile oil, and polysaccharides. Saponins, as the major chemical components of PNS, have been found to have promising anti-diabetic effects [9]. Dammarane-type triterpenoid saponins are the major bioactive saponins. They are composed of ginsenoside Rb1, Rg1, Rc, Rd, Re, Rf, Rh1, Rg, notoginsenoside R1 (NR1), etc. [10]. It was reported that ginsenoside Rb1 exerts significant anti-obesity, anti-hyperglycemic, and anti-diabetic effects by regulating glycolipid metabolism and improving insulin and leptin sensitivities. It also inhibits the JNK signaling pathway, and JNK1 and c-Jun expression in STZ-induced diabetic rats, resulting in negative regulation of the expression of the inflammatory molecules IL-6, IL-1β, and TNF-α [11]. Ginsenoside can promote insulin synthesis (Rb2), and increase insulin secretion (Rg3) of rat pancreatic β cells (INS-1) in a high-glucose environment [12] as well as inhibit β cell apoptosis through activating ERK and p38 MAPK phosphorylation. Other studies showed that PNS upregulate the expression of miR-181b, caspase-3 bcl-2 and activate PI3K-AKT-mTOR pathway, which blocks autophagy expression genes and autophagy membrane marker, thereby reducing apoptosis and autophagy [13]. However, compound-level studies can hardly account for the complex interaction networks of components and molecular biological systems in PNS, leaving a gap between the actual effects of PNS and our current knowledge [14]. While these studies showed evidence of diabetes-related biological pathways and gene networks, they do not provide a detailed representation of how PNS as a whole influence diabetes in pancreatic β cells.

MicroRNAs are a class of ~ 22 nucleotides noncoding RNAs that are post-transcriptional regulators of the gene expression of target genes. By interacting with complementary sites in the 3′ untranslated region of the target mRNAs [15], miRNA play gene-regulatory roles and produce many changes in physiological and pathological processes. Studies have shown that miRNAs have been involved in the regulation of different biological processes, including apoptosis, proliferation, metabolism, cellular differentiation, and gene regulation [16, 17]. Through sequencing by hybridization or sequencing by synthesis, NGS can sequence the transcriptome of desired samples efficiently and accurately [18]. However, relatively few studies implement NGS-based approach to elucidate the functions of Chinese herbs when discussing metabolic disorders such as diabetes treatment. In this study, we utilized NGS to carry out transcriptomic profiling for uncovering essential anti-oxidation-related genes, miRNAs and pathways, like others previously [19, 20]. Furthermore, it also aids in the identification of changes in diabetic-related pathways and key active compound in action under specific therapeutic treatment [21].

By adopting systems biology approaches that integrates transcriptomic data with miRNA interaction networks, we have significantly expanded our understanding of molecular pathways disrupted in metabolic diseases and prove helpful in identifying novel biomarkers, and disease mechanisms [22, 23]. In the context of metabolic disorders such as diabetes, network biology and network pharmacology have been used to unravel unique biological pathways. For instance, a network approach using transcriptomic data identified *Cylcocarya paliurus* treatment inhibited inflammation and apoptosis pathways [24]. Furthermore, Kutlu et al*.* constructed a genetic network of cytokine-regulated genes using time-course microarray data and revealed the gene networks activated in β cells under prolonged immune assault [25].

Since PNS has been known to be effective in treating diabetes, we wished to examine and explain the detailed effect of PNS in rat pancreatic β cells (INS-1). To refine our research, we investigated the genes involved in enriched pathways under PNS treatment, specifically the regulation of oxidative stress. With the knowledge from our miRTarBase database [26] to construct a miRNA-gene regulatory network and gene-target pathway network. Specifically, we examined the activity and expression of genes and proteins including Superoxide dismutase 1 (SOD1), heme oxygenase-1 (Hmox1), and glutamate cysteine ligase (GCLc) and results of our study suggest that PNS protects pancreatic β cells from oxidative stress.

## Methods

### Cell culture

Pancreatic β cells INS-1 (Cellcook Biotech Co., Ltd, Guangzhou city, China) were routinely cultured in RMPI-1640 (Gibco cat: 11875) media supplemented with 10% FBS, 1X sodium pyruvate and 0.05 mM β-mercaptoethanol and incubated at 37 °C in a humidified environment with 5% CO_2_. The cells were passaged and detached with Trypsin–EDTA.

### Metabolite extraction

100 mg *Panax notoginseng* (Efong Pharmaceutical) samples were thawed on ice, and metabolite were extracted with 0.5 mL of pre-chilled 80% methanol. The extraction mixture was then stored in 30 min at − 20 °C. Sample was then centrifuged at 20,000 × g for 10 min following vacuum drying of supernatants and redissolved with 100 μL 80% methanol. Samples were then stored at − 80 °C prior to the LC–MS analysis.

### Compound detection with UPLC-MS/MS

Ultimate 3000 UPLC (Thermo Fisher Scientific, Bremen, Germany) was utilized to carry out the chromatographic separation of all samples and ACQUITY BEH C18 column (2.1 × 100 mm, 1.7 µm, Waters, Milford, USA) was used for reverse phase separation. In this work, the flow rate was determined at 0.3 mL/min and the mobile phase consisted of solvent A (water, 0.1% formic acid) and solvent B (Acetonitrile, 0.1% formic acid). Gradient elution conditions were set as follows: 0–0.8 min, 2% B; 0.8–2.8 min, 2% to 70% B; 2.8–5.6 min, 70% to 90% B; 5.6–6.4 min, 90% to 100% B; 6.4–8 min, 100% B; 8–8.1 min, 100% to 2% B; 8.1–10 min, 2% B. The high-resolution tandem mass spectrometer Q-Exactive (Thermo Scientific), operated in both positive and negative ion modes, was used to detect metabolites eluted from the column. During the Q-Exactive analysis, precursor spectra with the range of 70–1250 m/z were collected at 70,000 resolutions to hit an AGC target of 3e6 with a maximum inject time of 100 ms. Addi-tionally, fragment spectra were collected at 17,500 resolutions to hit an AGC target of 105 with a maximum inject time of 50 ms. A top 3 configuration to acquire data was set in DDA mode and results are available in Additional file 19: Table S9. Parallel reaction monitoring (PRM) mode was used for the content analysis of selected compounds. Raw compound data were then analyzed with Skyline software [27] and XcaliburTM 4.1 software (ThermoFisher, San Jose, CA, USA, 2019) for peak extraction and metabolite identification. The secondary mass spectrogram information in the sample experiment was then used to match with standard databases including MzCloud (Thermo), Traditional Chinese Medicine (Thermo), BMDMS-NP (BMDMS), Vani-ya-Fiehn_Natural_Products (Fiehn lab), PlaSMA (RIKEN). The matching error settings were 0.01 Da for the first level, 0.05 Da for the second level, and a matching score > 70 was considered a reliable metabolite.

### PNS preparation, dosage sensitivity assays and treatment conditions

Prepare 1 mL of PNS solvent by dissolving in RMPI-1640 media and centrifuge at 12,000 rpm for 5 min to obtain supernatant and dilute solvent into 8 different concentrations in total (1 g/mL, 3.3 × 10^–1^ g/mL, 10^–1^ g/mL, 3.3 × 10^–2^ g/mL, 10^–2^ g/mL, 3.3 × 10^–3^ g/mL, 10^–3^ g/mL and 3.3 × 10^–4^ g/mL). The dosage sensitivity assay was performed to acquire inhibition of cell viability when INS-1 cells are treated with PNS to generate dose response curve. Experiments were carried out in sextuplicate. In short, INS-1 cells were seeded at 5 × 10^3^ cells/well into 96-well microplates and incubate for 24 h before replacing clean media with different concentrations of PNS solvent with 8 different concentrations, 100 µL/well. After 24 h of incubation, CCK-8 assay was performed by removing the PNS solvents and adding 100 µL/well of CCK-8 reagent, incubating for 1.5 h and reading absorbance of each well at 450 nm in a microplate reader. Inhibition ratio (IR) was then calculated used to decide upon three PNS concentrations used (Low: 11.2, Medium: 20.2 and High: 29.1 mg/mL).

### RNA isolation

For RNA isolation, INS-1 cells were plated at a density of 10^5^ cells per 10 cm dish and incubated in RPMI-1640 for 24 h before exchanging with media containing PNS at 3 different concentrations and a model control with 2 experimental repeats (different cell generations) for the 4 conditions. On the following day, the cells were collected with 1 mL TRIzol reagent (Invitrogen) per dish and stored at − 80 °C before RNA extraction. mRNA was extracted from INS-1 cells using standard RNA isolation TRIzol protocol and miRNA extracted with miRNeasy Mini Kit (Qiagen, Hilden, Germany). Extracted RNA was then dissolved in RNase-free water and stored at − 80 °C before further treatment. Concentration of RNA was measured using Qubit Fluorometric Quantification (ThermoFisher, Waltham, MA, USA).

### Library preparation for RNA and small RNA sequencing

RNA-seq and small RNA-seq cDNA libraries were constructed following the MGI mRNA library preparation assay (Hieff NGS^®^ Ultima Dual-mode RNA Library prep kit for MGI, 13333ES96) protocol, previously purified and fragmented with Hieff NGS^®^ mRNA Isolation Master Kit (Hieff NGS^®^, Cat#12603). Resulting cDNA library fragments were validated using Agilent 2100 Bioanalyzer (Agilent) and cDNA libraries were measured using quantitative reverse-transcription PCR (qRT-PCR) (Roche, LightCycler^®^ 480 system, Basel, Switzerland) as well as Qubit fluorometer (Invitrogen, Carlsbad, California, USA). Then they were pooled, and sequenced on BGI NGS platform (DNBSEQTM-T7), pair-ended mode with 150 bps. Coverage depth were approximately 20 million and 5 million reads obtained in RNA-seq and miRNA-seq, respectively.

### RNA sequencing data analysis

Raw data was processed by nf-core/rnaseq pipeline (v3.0) with the parameter of “--genome Rnor_6.0 --gencode” to get the read count matrix for all samples. For details, low quality and adaptor sequences in raw reads were trimmed using Trim Galore. After removal of ribosomal RNA by SortMeRNA, the cleaned reads were aligned to the reference genome (Rnor_6.0, UCSC) using STAR. iDEP (http://ge-lab.org/idep/), an integrative web application, was used to perform differential expression analysis. mRNA count matrix was first preprocessed to remove the low level expressed genes with count less than 4 in all samples and then normalized to log2-CPM matrix. PCA, bar chart and distribution plot were used to visualize the data quality. Differential expressed mRNA analysis was performed with DESeq2 with a False Discovery Rate (FDR) ≤ 0.05 and fold change ≥ 1.5.

### Small RNA sequencing data analysis

Small RNA-seq raw data was inputted in nf-core/smrnaseq pipeline (v1.0.0) with the parameter of “--genome Rnor_6.0”. Firstly, low quality and adaptor sequences in raw reads were trimmed using Trim Galore. Secondly, the cleaned reads were aligned against miRbase [28] mature miRNA and hairpin using Bowtie. Finally, a list of top expression hairpin and mature miRNA after TMM normalization was obtained using edgeR [29]. MiRNA CPM matrix was first preprocessed to remove the low level expressed miRNA with CPM less than 10 in all samples. Correlation matrix was used to analysis the data quality. Differential expressed miRNA analysis was performed by small program with *p*-value ≤ 0.05 and fold change ≥ 8.

### A computational approach for reconstruction of miRNA gene regulatory network

The mature DEmiRs were used to analyze the target genes. Experimental MTIs were obtained from miRTarBase [26]. The predicted MTIs were obtained using two prediction databases. MiRWalk 2.0 [30], which integrated several MTI prediction databases, was used to predict the target gene of DEmiRs by TarPmiR algorithm. All 13 parameters in this algorithm were optimized automatically. MiRDB [31] was also used to predict the MTI with default parameters. We select the DEGs which was detected MTI relation with DEmiRs to construct miRNA regulatory network. Cytoscape software was used to visualize the MTI network of up and down regulated DEGs separately. To discuss the function of the MTI network, we further analyzed the target up and down DEGs functional annotation by using MetaScape [32] separately.

### Network construction and analysis of DEGs related pathways

Metacore (Clarivate MetaCore + MetaDrug™ version 20.4 build 70300) was used to analysis the up and down DEG related pathways and GO processes. The parameters were set as *p*-value ≤ 0.05 and FDR ≤ 0.05. After we got the enriched pathways, we selected the significant pathways and related DEGs for further discussion. Cytoscape software was used to visualize the DEG-pathway topology network.

### Quantitative real-time PCR

The expressions of specific target genes will be determined. Total RNA of TCMs treated INS-1 cells were extracted, and the RNA was reverse transcribed into cDNA by reverse-transcription polymerase chain reaction. Quantitative real-time PCR (QuantStudio 6 Flex Real-Time PCR System) with the SYBR green system (PowerUP SYBR green, Applied Biosystems) were used to determine the relative gene expression levels in cells compared to control. MiRNA was reverse transcribed using miRcute Plus miRNA First Strand cDNA Synthesis Kit (Tiangen Biotech, Beijing, China) and amplified with SYBR green system (PowerUP SYBR green, Applied Biosystems). The expressions were analyzed with housekeeping genes glyceraldehyde-3-phosphate dehydrogenase (GAPDH) and U6 for RNA and miRNA, respectively. All primers used are listed in Table 1.

**Table 1:** Sequences of primer pairs of mRNA and miRNA used in the real-time quantitative PCR reaction

Gene symbol	Forward (5′–3′)	Reverse (5′–3′)
Hmox1	ACCGTGGCAGTGGGAATTTA	CAAGATTCTCCCCTGCAGAGA
GCLc	TGAGTCTCCTCTTGCTGTGTACGT	GGGACTGCTCTGCAAAGGAA
Txn1	GTGGTGTGGACCTTGCAAAA	CTGGCAGTCATCCACGTCTACTT
SOD1	CACTGCAGGACCTCATTTTAATCC	GTCCTTTCCAGCAGCCACAT
PRDX1	AGCTCGACTCTGCTGATAGCAA	AATTGTCCATCGGGCATAACA
Lpl	GCTCCATCCATCTCTTCATTGAC	AGGCAGAGCCCTTTCTCAAAT
ABCG1	TGGAGAACGCGAAGCTATACC	CAGCCCGGATTTTGTATCTCA
Cav-1	CCATGGCAGACGAGGTGAAT	ACCACGTCGTCGTTGAGATG
RPS6	GAAAGCCCTTAAACAAAGAAGGTAAG	ATACGTCGGCGTTTGTGTTG
Gab1	TCCCACCACACCCAGACACT	TGGGCTCTGGTGGGTTCA
GAPDH	GGTGGACCTCATGGCCTACA	CAGCAACTGAGGGCCTCTCT

MiRNA	Forward (5′–3′)	Reverse (5′–3′)
Rno-miRNA-139-5p	GTCTACAGTGCACGTGTC	CCAGTTTTTTTTTTTTTTTCTGGAG
Rno-miRNA-24-3p	AGTGGCTCAGTTCAGCA	CCAGTTTTTTTTTTTTTTTCTGTTCCT
Rno-miRNA-671	GCAGTCCGGTTCTCAG	GGTCCAGTTTTTTTTTTTTTTTGGT

### Dual-luciferase reporter assay

The 3′-UTR of GCLc and SOD1 genes carrying the predicted binding sites of miR-139-5p and miR-24-3p (http://mirwalk.umm.uni-heidelberg.de/) was synthesized into the pmirGLO vector (E1330, Promega Corporation) to generate wild-type (WT) and mutant (MUT) GCLc and SOD1 dual-luciferase expression vectors (SOD1-WT, SOD1-MUT, GCLc-WT, and GCLc-MUT). 293T cells were co-transfected with SOD1-WT or SOD1-MUT or GCLc-WT or GCLc-MUT, and miR-24-3p or miR-139-5p or the control mimics using Lipofectamine^®^ 2000 (Invitrogen). After 24 h, the activities of Firefly and Renilla luciferase were determined using Dual-Luciferase Reporter assay system (Promega Corporation). The results were conducted in triplicates and shown as the ratio of Renilla to Firefly luciferase activity.

### Western blotting

For the protein expression analysis of SOD1, Hmox1 and GCLc proteins, PNS treated INS-1 cells were washed with ice-cold phosphate buffer saline (PBS), collected and lysed using RIPA lysis buffer (P0013C, Beyotime, China) supplemented with a protease inhibitor PMSF (ST506, Beyotime, China) according to manufacturer’s protocol to obtain total protein. Equal amounts of protein lysates were resolved in 10% SDS-PAGE (SK6010-250, Coolaber, China), transferred on the Immobilon-PSQ PVDF blotting membrane (ISEQ00010, Merck) using wet transfer. Membranes were incubated in 5% BSA (ST023-200 g, Beyotime, China) in Tris-buffered saline pH 7.6 containing 0.1% Tween-20 following primary antibody incubation overnight. Antibodies used against SOD1 (dilution: 1:50000, ab51254), Hmox1 (dilution: 1:50000, ab68477), GCLc (dilution: 1:20000, ab190685) were purchased from Abcam. GAPDH (dilution: 1:100000, 60004-1-Ig) was purchased from Protein Tech. Membranes were then washed with TBST and incubated with secondary antibody Anti-Mouse IgG, HRP-linked antibody (A0208, Beyotime, China) and Anti-Rabbit IgG, HRP-linked antibody (A0216, Beyotime, China). Protein detection was performed using ECL western blotting substrate (SL1350, Coolaber) and captured with Amersham Imager 600 (GE Healthcare Life Sciences). Expression of GAPDH was used as a control.

### Compound target protein prediction

The pathway interpreter, a credited biology pathway information-based algorithm, was utilized to find possible pathways of protein targets to DEGs. It mainly includes two steps: compound target protein prediction and functional enriched pathway network prediction. The metabolites of PNS were first annotated with PubChem id and corresponding SMILES structure as shown in Additional file 20: Table S10. Then, we predicted the target protein of PNS metabolites with DeepConv-DTI, SEA, and SuperPred and selected targets with both accuracy and model probability over 0.9 where results are available in Additional file 21: Table S11. DeepConv-DTI is a deep learning approach to predict small compound drug-target interactions [33]. We trained the model with an in-house drug-target interaction dataset collected from KinaseSARfari, PubChem BioAssay database, DrugBank, and IUPHAR, which included over 100,000 experimentally validated drug-target interactions. We selected high scoring predicted target proteins for major components of PNS compounds. SEA is a well-known similarity-based target prediction method [34]. We selected targets with *p*-value less than 1 × 10^–12^. SuperPred is a drug target prediction webserver [35].

### Network discovery between PNS target proteins and DEGs

To understand the molecular mechanisms of PNS activating oxidative stress reaction, we investigated the possible pathway between PNS targets and DEGs with an in-house pipeline called pathway interpreter. Most pathway analyses can only give general predictions, just arbitrary combinations of current pathway information from mixed sources. The pathway interpreter was designed to give credited pathway prediction with a biology knowledge-based algorithm.

The pathway interpreter gives Impact Factor (IF) based reliability scores for all interactions in the pathway for quality control. The oxidative stress reaction related pathway data were collected from Reactome [36], MetaCore [37], and KEGG [38] databases. The reference information is collected simultaneously. Based on the IFs of collected references, the IF score is calculated for creditability check. The exponential algorithm is applied for IF score calculation to avoid bias from the accumulation of low IF references. $IF score= \sum \left(\frac{{e}^{IF / 5}}{2}-\frac{1}{2}\right)$. The reaction-wise IF scores and general statistics for the IF distribution of target biology pathways were calculated for further evaluation.

The pathway interpreter found all the possible pathways starting from the PNS target proteins to the DEGs with step lengths less than 11 in the oxidative stress reaction process. The biology-based assessment score is calculated for all the pathways to filter out the top 10 reasonable paths for every target-DEG combination (Additional file 22: Table S12). The score calculation is based on the following biology principles: disfavor the extension of steps (*Penalty* = − n(n + 1)) for the length of n) and favor key molecules in signal transduction (No penalty for centroid molecules). The first 8 PNS targets were selected to construct the predictive pathway with 17 nodes, results are shown in Fig. 8a.

### In vivo experimental settings of PNS and ginsenoside Re protective functions

In the in vivo experiment, we conducted anti-oxidative function validation in two models of zebrafish (WT and hyperglycemia model) based on Reactive Oxygen Species (ROS) level, glucose level and gene expression analysis of anti-oxidation related genes including superoxide dismutase 1 (SOD1), heme oxygenase-1 (Hmox1), glutamate cysteine ligase (GCLc), lipoprotein lipase (Lpl), caveolin-1 (CAV-1), Kelch-like ECH-associated protein 1 (Keap1) and nuclear factor erythroid 2-related factor 2 (Nrf2). The zebrafish experiment was assisted by Hangzhou Hunter Biotechnology, Inc. and was accredited by the International Association for Assessment and Accreditation of Laboratory Animal Care (AAALAC, 001458) and was licensed to use experimental animals (SYXK [Z] 2022-0004). Experimental dose of PNS and ginsenoside Re for in vivo evaluation is available in Additional file 22: Table S12 and further experimental designs and results are available in Additional file 25: Material and methods.

### Statistical analysis

All statistical analyses were performed using the SPSS 16.0 software (SPSS, USA), and *p*-value < 0.05 was considered statistically significant. For quantitative analysis, all data were presented as mean ± SE, and results were statistically compared between TCM-treated and control zebrafish groups.

## Results

**Fig. 1:**
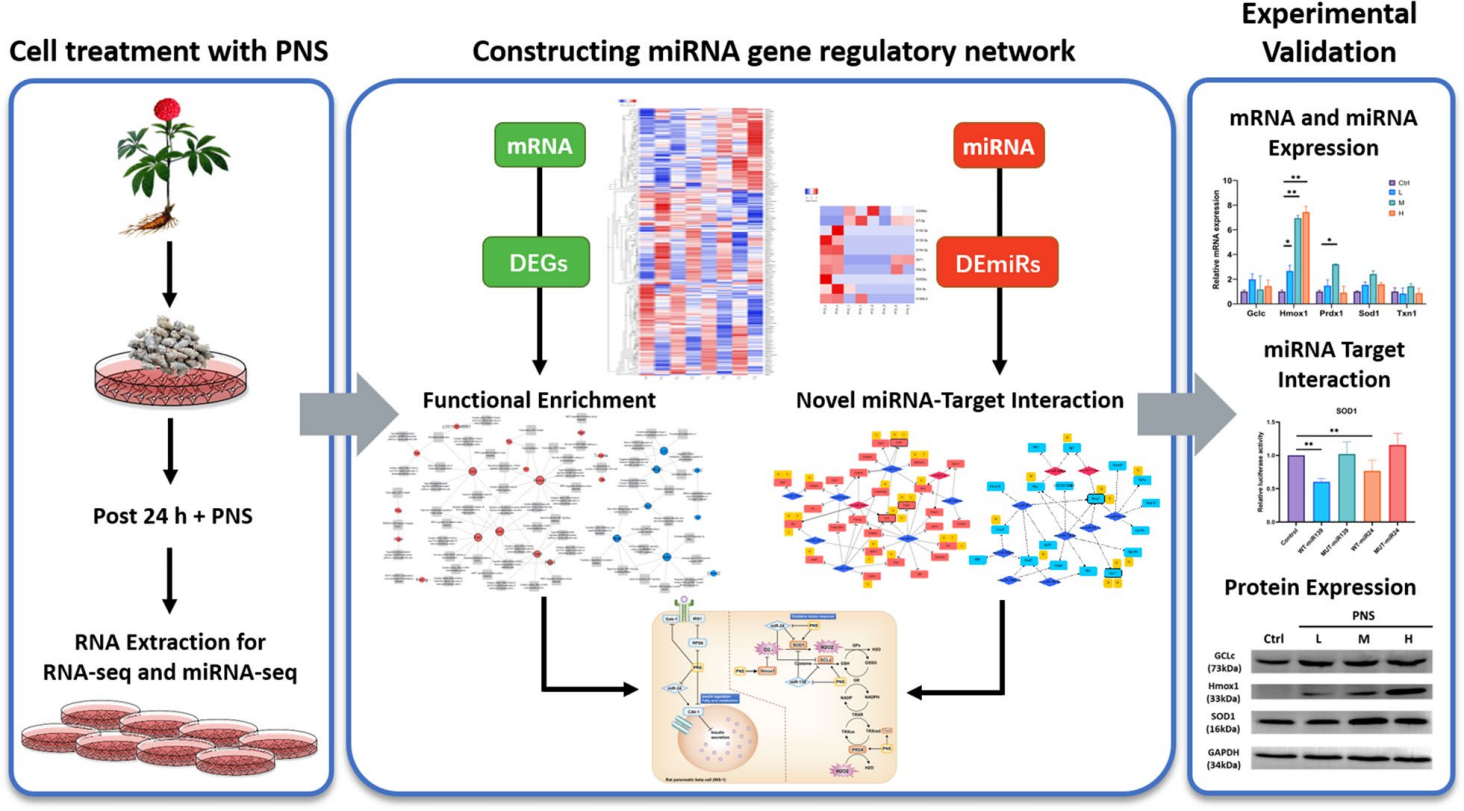
Schematic representation of the study workflow to identify the regulatory network of PNS in treating metabolic disorders

Many PNS compounds are known to be able to protect against metabolic disorders such as diabetes. However, we wanted to explore the detailed role and mechanism of its protective effect as a whole mixture in pancreatic β cells (INS-1) and uncover novel miRNA target relations. A schematic overview of the experimental and computational framework to identify the regulatory effect of PNS treatment is presented in Fig. 1, where INS-1 cells were treated with a number of different PNS concentrations to obtain three administration concentrations (11.2, 20.2 and 29.1 mg/mL). RNA was then extracted for analysis of high-throughput expression profiles. Quality control filtered out the noise data to obtain clean RNA-seq and miRNA-seq data. Computational methods including the identification of differentially expressed genes (DEGs) and differentially expressed miRNAs (DEmiRs) and constructing regulatory network and functional annotation of target genes were utilized. The raw sequencing data of PNS treated samples against control models are listed in Additional files 11, 12 and 13: Tables S1, S2 and S3.

### Effect of different PNS concentration treatments on pancreatic β cell growth

After exposure to three PNS concentrations for 24 h, pancreatic β cell viability was measured and showed to be insignificant up to condition of log(− 2) g/mL, and results are shown in Fig. 6a. Cell morphology and growth patterns of pancreas β cells were observed under a microscope interstingly, showed increase in growth under 20.2 mg/mL and 11.2 mg/mL PNS treatments compared to control and 29.1 mg/mL PNS concentration. Cell morphology results are shown in Fig. 6b.

### Effect of different PNS treatments on gene expressions in pancreatic β cell

**Fig. 2:**
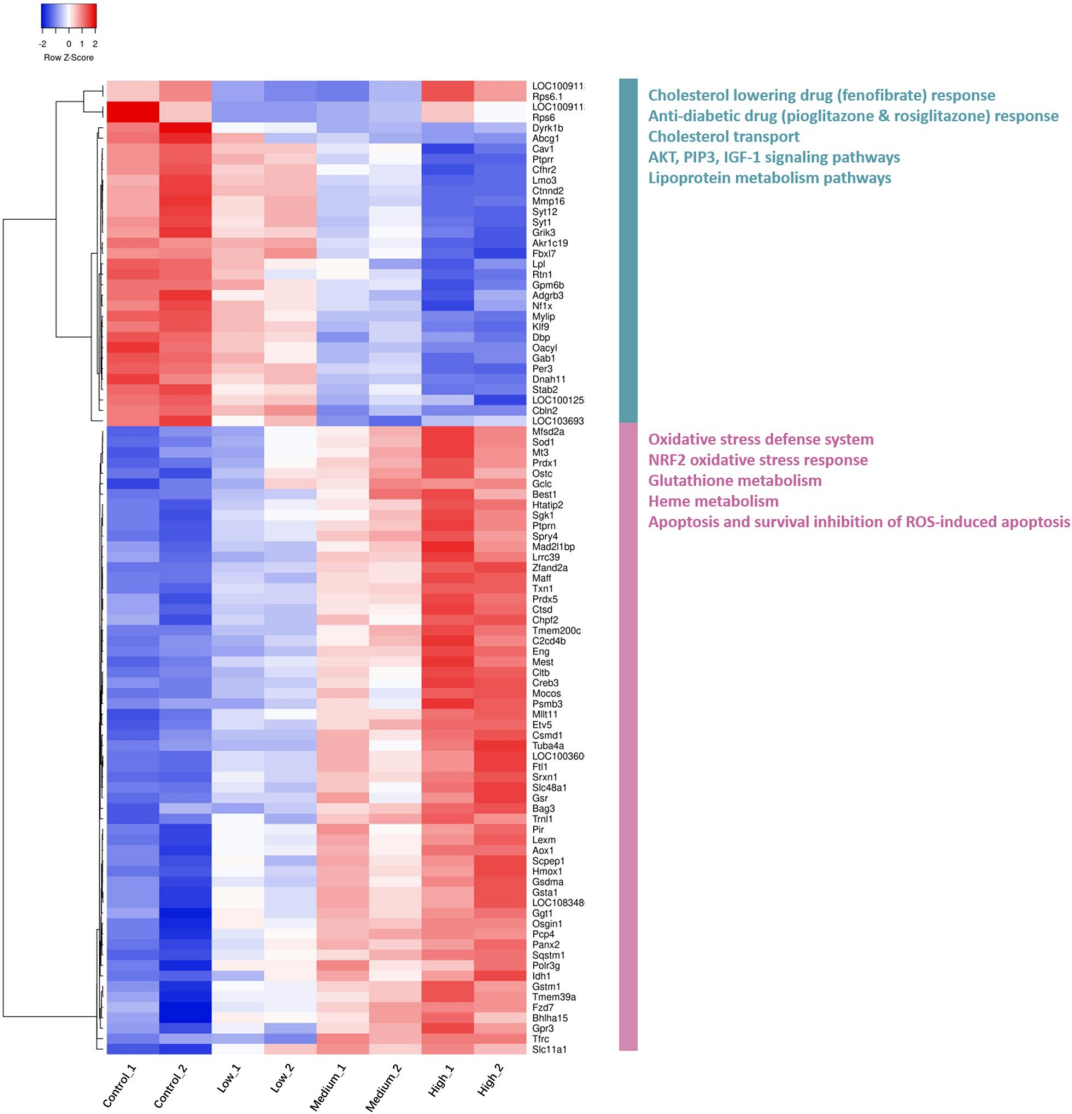
Heatmap presents expression values of Differentially Expressed Genes (DEGs) in PNS-treated pancreatic β cells compared to the control model. DEGs that were seen across 2 or 3 PNS concentrations were taken into account, resulting in 63 up-regulated DEGs and 33 down-regulated DEGs. Up-regulated DEGs (pink label) were functionally enriched in nuclear factor erythroid-derived two signaling, glutathione metabolism, and cellular iron ion homeostasis related pathways, while down-regulated DEGs (turquoise label) were functionally enriched in organic hydroxy compound transport, negative regulation of ERK1 and ERK2 cascade, and cholesterol homeostasis related pathways. (Fold change ≥ 1.5, False Discovery Rate (FDR) ≤ 0.05). Low: 11.2, Medium: 20.2 and High: 29.1 mg/mL

To understand the molecular mechanism of PNS, we investigated the transcriptome profiling of pancreatic β cells and filtered out the DEGs present across two or three PNS concentrations (Fig. 2). In our study, there were eight RNA samples under three increasing PNS concentrations (11.2, 20.2 and 29.1 mg/mL), one control model and two experimental repeats (different cell generations) for each sample group (Additional files 11 and 12: Table S1 and S2). Read quality of RNA-seq and miRNA-seq are shown in Additional file 1: Fig. S1. Low expression genes were removed and count matrix normalized, resulting in around 14,000 genes for analysis across eight samples. Then principal component analysis (PCA) was conducted for pre-processed mRNA-seq matrix and showed clearly defined groups between the different concentrations that were similar across two experimental repeats (Additional file 1: Fig. S1A and S1B).

The volcano plots in Additional file 2: Fig. S2 presented the DEGs, acquired from RNA-seq data after cleaning up and normalizing the raw sequencing data. Detailed information on all DEGs (mRNA log2-CPM matrix) are provided in Additional file 14: Table S4. In pancreatic β cells, 14,453 genes (count > 4 and converted to log2-CPM matrix) were detected and DEGs of each concentration were determined separately; relative to the model control group. Expression values exceeding 1.5-fold change with FDR ≤ 0.05 were included for further analysis. Number of up-regulated DEGs were 8, 63 and 381 respectively to 11.2, 20.2 and 29.1 mg/mL PNS concentration. 63 DEGs overlapping two or three concentrations (Additional file 4: Fig. S4) were selected. The number of down-regulated DEGs were 3, 35 and 428; 33 common DEGs were found to overlap two or three concentration conditions. Genes related to insulin are shown in Additional file 5: Fig. S5 and around half of our entire mRNA matrix presented positively or negatively correlated gene expression with PNS. In contrast, over half of the insulin-related genes of our mRNA matrix showed no obvious correlation.

### Effect of PNS concentration on miRNAs expression in pancreatic β cells

MiRNA-seq was conducted as well to investigate the miRNA expressions under PNS influence. The detailed information of DEmiR raw data is provided in Additional file 13: Table S3. In pancreatic β cells, a total list of 260 miRNAs (count > 4 and converted to CPM [count per million reads mapped]) were detected (Additional file 15: Table S5) and DEmiRs of each PNS treated concentration (11.2, 20.2 and 29.1 mg/mL) were determined separately, relative to model control. Only miRNAs with fold change exceeding eight for both down and up-regulation (False Discovery Rate (FDR) ≤ 0.05) were considered. Low expression miRNAs were again removed and count matrix normalized. MiRNAs that were differentially expressed across two or three PNS concentration groups were selected and filtered with miRWalk (high-performance prediction database) [30] and miRDB (support prediction database) to remove hairpins [31], providing us with a final list of 43 up and down-regulated DEmiRs, 14 up-regulated and 29 down-regulated across three PNS concentrations. After inputting the list into miRTarBase (experimentally validated targets) [26], our DEmiRs showed no experimentally validated relation to our DEGs (Additional file 7: Fig. S7), suggesting novel miRNA Target Interactions (MTIs).

**Fig. 3:**
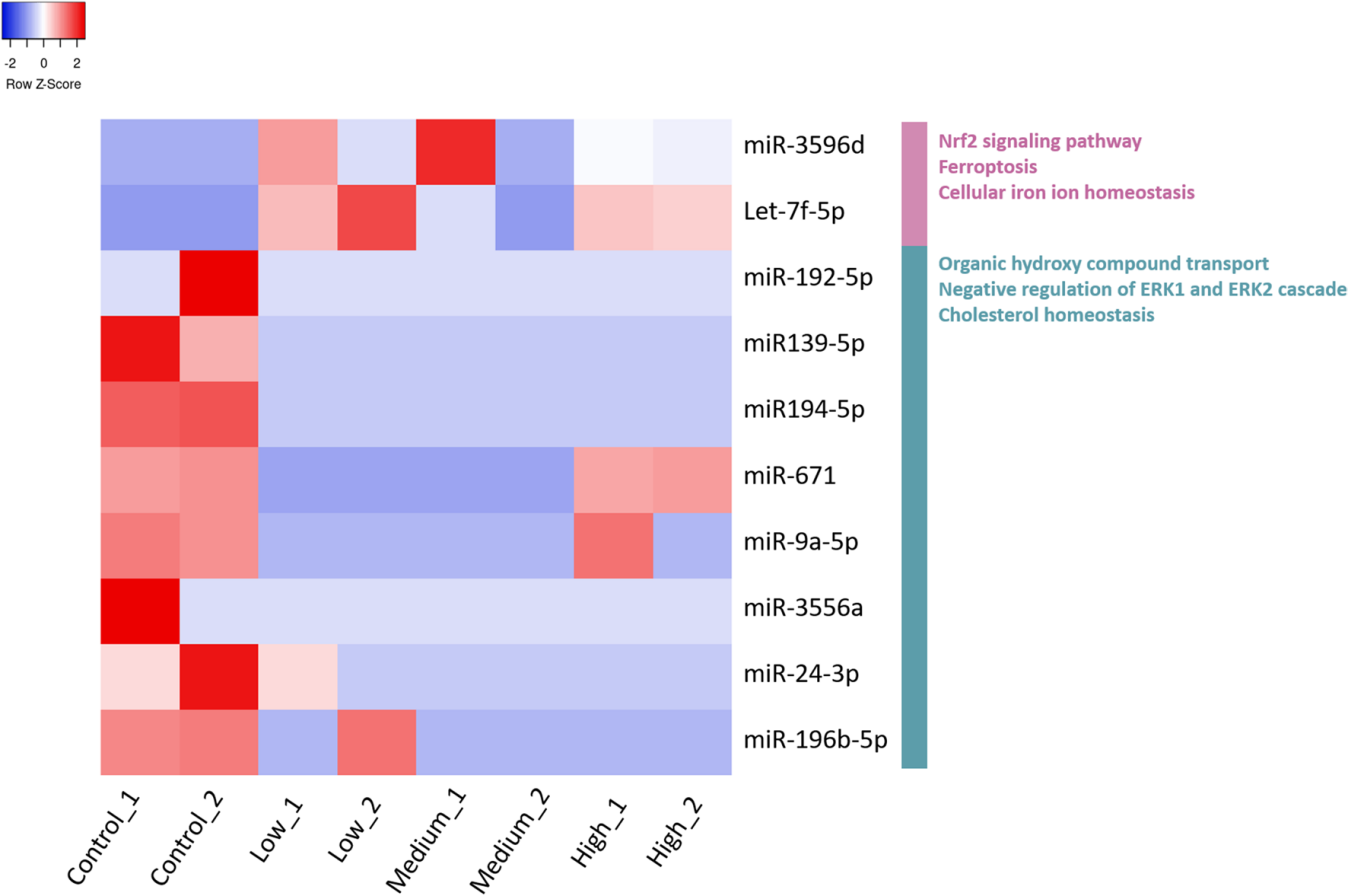
Heatmap presents expression values of Differentially Expressed miRNA (DEmiR) of PNS treated pancreas β cells (Fold change ≥ 8, *p*-value ≤ 0.05). Low: 11.2, Medium: 20.2 and High: 29.1 mg/mL

Only mature miRNAs excluding hairpin candidates that were present across two or three PNS concentration treatments were qualified candidates and these conditions left two up-regulated (miR-3596d, and let-7f-5p) and seven down-regulated (miR-139-5p, miR-194-5p, miR-671, miR-9a-5p, miR-3556a, miR-24-3p and miR-196b-5p) DEmiRs. Six of which were found to influence DEGs related to diabetes from our gene analysis. Some miRNA expressions were significantly different post PNS treatment, such as miR-3596d and let-7f-5p being unevenly up-regulated in the PNS-treated groups com-pared with the control group across three concentrations (Fig. 3). Volcano plot graphs in Additional file 3: Fig. S3 presents the DEmiRs for PNS concentrations 11.2 mg/mL, 20.2 mg/mL and 29.1 mg/mL were 6, 4 and 10, respectively. Number of down-regulated DEmiRs for L, M and H groups were 16, 16 and 10, respectively.

### Transcriptome and functional enrichment analysis demonstrated that PNS influences pancreatic β cell by protecting against oxidative stress damage

**Fig. 4:**
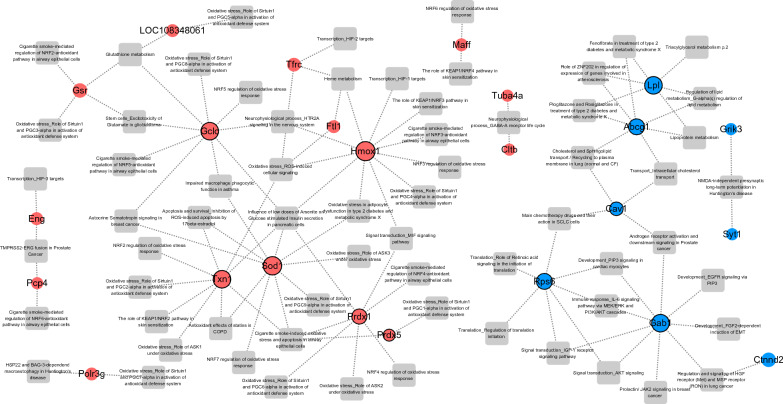
Relationships between DEGs of pancreatic β cells treated with PNS in comparison with control model group and their related biological pathways retrieved from MetaCore. Red circle nodes represent up-regulated DEGs; blue circle nodes represent down-regulated DEGs and grey squares are the functionally enriched pathways. GCLc, SOD1, Txn1, Hmox1, PRDX1, LPL, ABCG1, CAV-1, RPS6, and GAB1 have higher degrees. The number of edges of each node is more significant than other DEGs (9 in GCLc, 10 in SOD1, 9 in Txn1, 11 in Hmox1, 7 in PRDX1, 6 in LPL, 7 in Abcg1, 4 in CAV-1, 7 in RPS6, and 9 in Gab1)

To understand the molecular mechanisms of PNS in metabolic disorders, we further investigated the transcriptome profiling across three different concentrations. GO and biological pathways for 96 DEGs in PNS treatment (63 up-regulated and 33 down-regulated) were predicted through MetaCore (Clarivate MetaCore + MetaDrugTM version 20.4 build 70,300) to identify the relationship between expressions of mRNAs in alleviating oxidative stress. Identification of the expected correlation through pathway analysis produced a final list of 138 and 142 pathways for up-regulated DEGs and down-regulated DEGs, respectively. This list was filtered and narrowed down to focus on related pathways incorporating at least 2 DEGs from our DEG data. 25 top enriched pathways for up and down-regulated DEGs were filtered out to generate a pathway network, and outliers were removed (Fig. 4). Up-regulated DEGs were functionally enriched in oxidative stress, NRF2-antioxidant response pathway, glutathione metabolism, heme metabolism and apoptosis and survival inhibition of ROS-induced apoptosis pathways (Fig. 2 and Additional file 16: Table S6). Down-regulated DEGs were functionally enriched in cholesterol-lowering drug (fenofibrate) response, anti-diabetic drug (pioglitazone and rosiglitazone) response, cholesterol transport, AKT, PIP3, IGF-1 signaling pathways and lipoprotein metabolism pathways (Fig. 2 and Additional file 17: Table S7). Therefore, MetaCore pathway analysis suggested that PNS treatment exhibited anti-diabetic effects through reduced oxidative stress response, glutathione metabolism, antidiabetic drug-related response, insulin signaling and reduced ROS-induced cell apoptosis.

GO clustering analysis of MTI related DEGs showed functional enrichment in NRF2 signaling pathway, ferroptosis, cellular iron ion homeostasis, hydroxy compound transport pathway, ERK1 and ERK2 cascade and cholesterol homeostasis related pathways (Additional file 6: Fig. S6). The results suggested that our DEmiRs have similar anti-diabetic effects compared to our DEGs under PNS treatment in INS-1 cells through involvement with oxidative stress response pathway and insulin regulation, and fatty acid metabolism.

### MiR-139-5p and miR-24-3p regulates oxidative stress response by targeting GCLc and SOD1

**Fig. 5:**
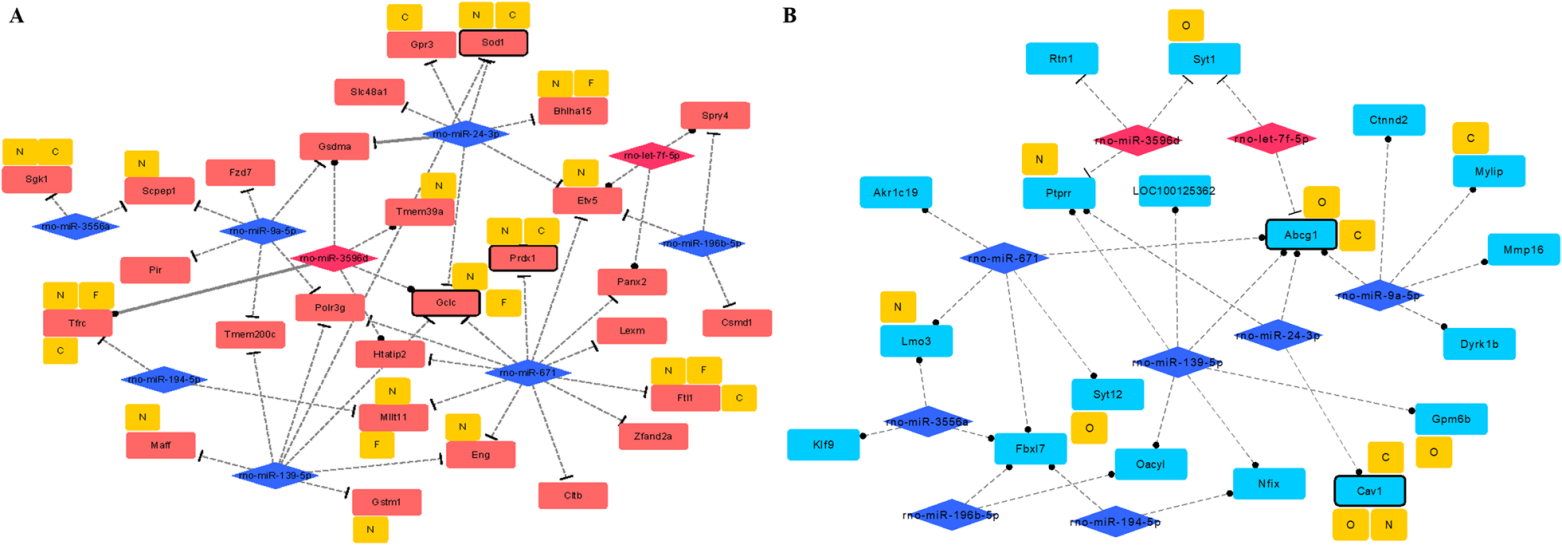
Predicted up and down-regulated DEG-related MTI network. **A** The annotation label “N” represents NFE2L2 pathway, “F” represents Ferroptosis, and “C” represents cellular iron ion homeostasis. **B** “O” represents organic hydroxy compound transport, “N” represents negative regulation of ERK1 and ERK2 cascade, and “C” represents cholesterol homeostasis. The rectangle denotes mRNA, the rhombus denotes miRNA (blue: down-regulated; pink: up-regulated). Dashed line: target prediction from 1 database. Grey line: target prediction from 2 databases. Rectangles with black rim: DEGs found in our highly enriched functions

To specifically understand the roles of our DEmiRs in the oxidative stress pathway discussed earlier, we constructed a miRNA-gene target regulatory network for DEmiRs and DEGs. miRNA-seq are susceptible and accurate tools for measuring miRNA expression under different environmental conditions. Often, we would assume miRNAs act as negative regulators of target mRNAs, so miRNA and target genes should present an inverse expression profile. To obtain reliable MTI pairs from our high throughput data, we used predicted evidence of miRNA and its target gene from miRNA databases. Nine miRNAs and targets genes were observed and a network connection was constructed (Fig. 5). For up-regulated DEG targets, miR-9a-5p/671/24-3p/139-5p and their target genes were hub nodes in the network (Fig. 5a), for down-regulated DEG targets, miR-9a-5p/139-5p/671 were hub nodes (Fig. 5b). Interestingly, miR-139-5p was predicted to suppress both SOD1 and GCLc gene expression, whilst miR-24-3p was predicted to suppress SOD1 and GCLc. Abcg1 gene, a main hub gene in the down-regulated DEG related MTI network, showed five miRNA regulators (miR-9a-5p/24-3p/139-5p/671 and let-7f-5p). The related GO analysis of each DEmiR with its related DEGs is also annotated in the MTI network. Up-regulated DEG, DEmiR enriched pathways include, “N” for NFE2L2 pathway, “F” for Ferroptosis, and “C” for cellular iron ion homeostasis, whilst for down-regulated DEG, DEmiR enriched pathways include, “O” for organic hydroxy compound transport, “N” for negative regulation of ERK1 and ERK2 cascade, and “C” for cholesterol homeostasis.

### The effect of PNS on oxidative stress related miRNA and gene expressions in INS-1 cells

**Fig. 6:**
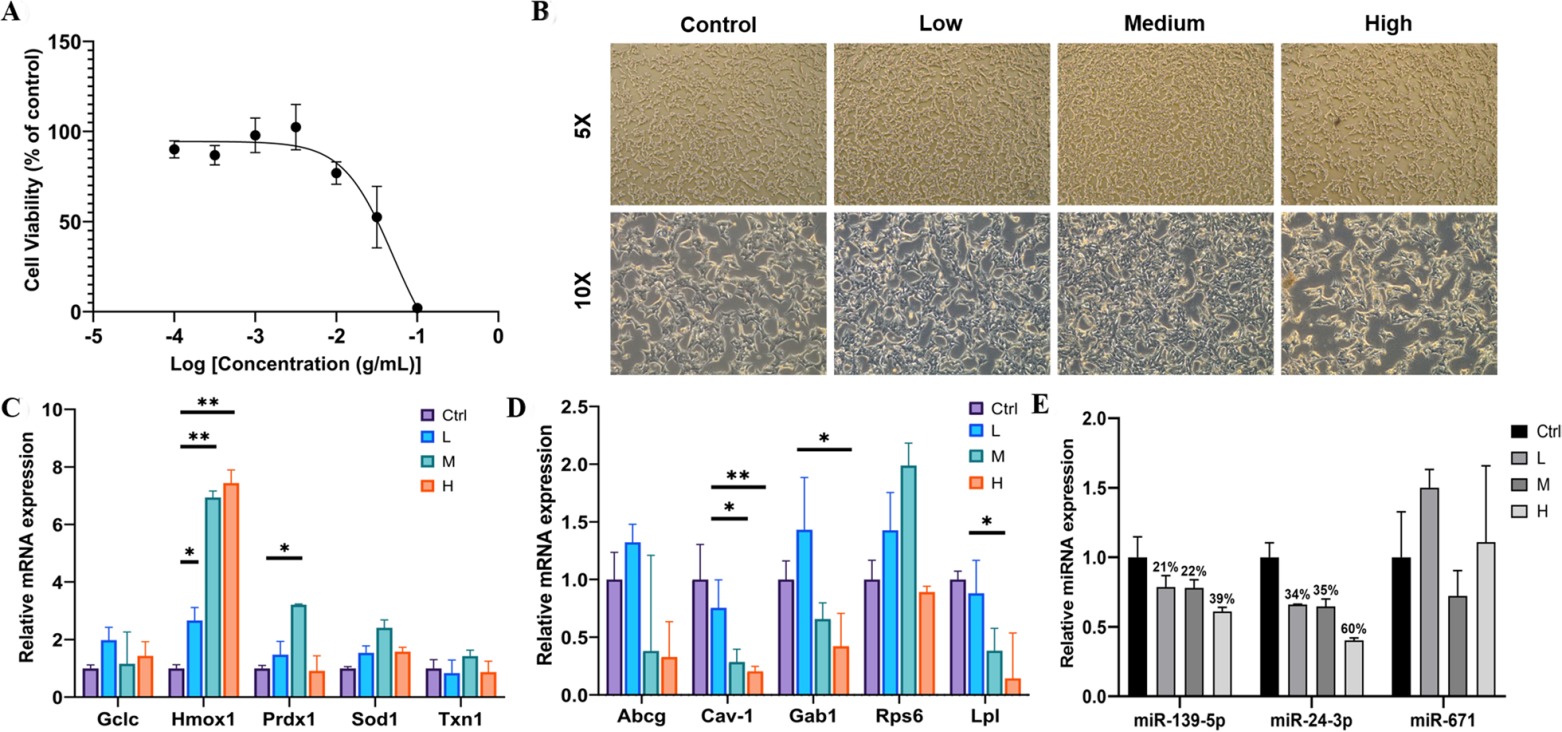
Effect of PNS treatment in INS-1 cell line. **A** Pancreatic β cell viability test determined with CCK-8 assay. Each point shows inhibition ratio of PNS on cells adjusted with control against a concentration gradient. **B** Cell morphology at different magnification post 24 h PNS treatment in 3 different concentrations, low (L, 11.2 mg/mL), medium (M, 20.2 mg/mL) and high (H, 29.1 mg/mL). Cells were collected after 24 h PNS treatment for RNA and miRNA sequencing. **C** Relative quantification (RT-qPCR) of up-regulated DEGs and **D** down-regulated DEGs transcripts. The gene encoding Glyceraldehyde 3-phosphate dehydrogenase (GAPDH) was used for normalization. **E** RT-qPCR of miRNA expression profiles. U6 was used for normalization. Each column represents the mean of three independent samples of different cell passage. * indicate significant differences between groups *p < 0.05; **p < 0.01

**Fig. 7:**
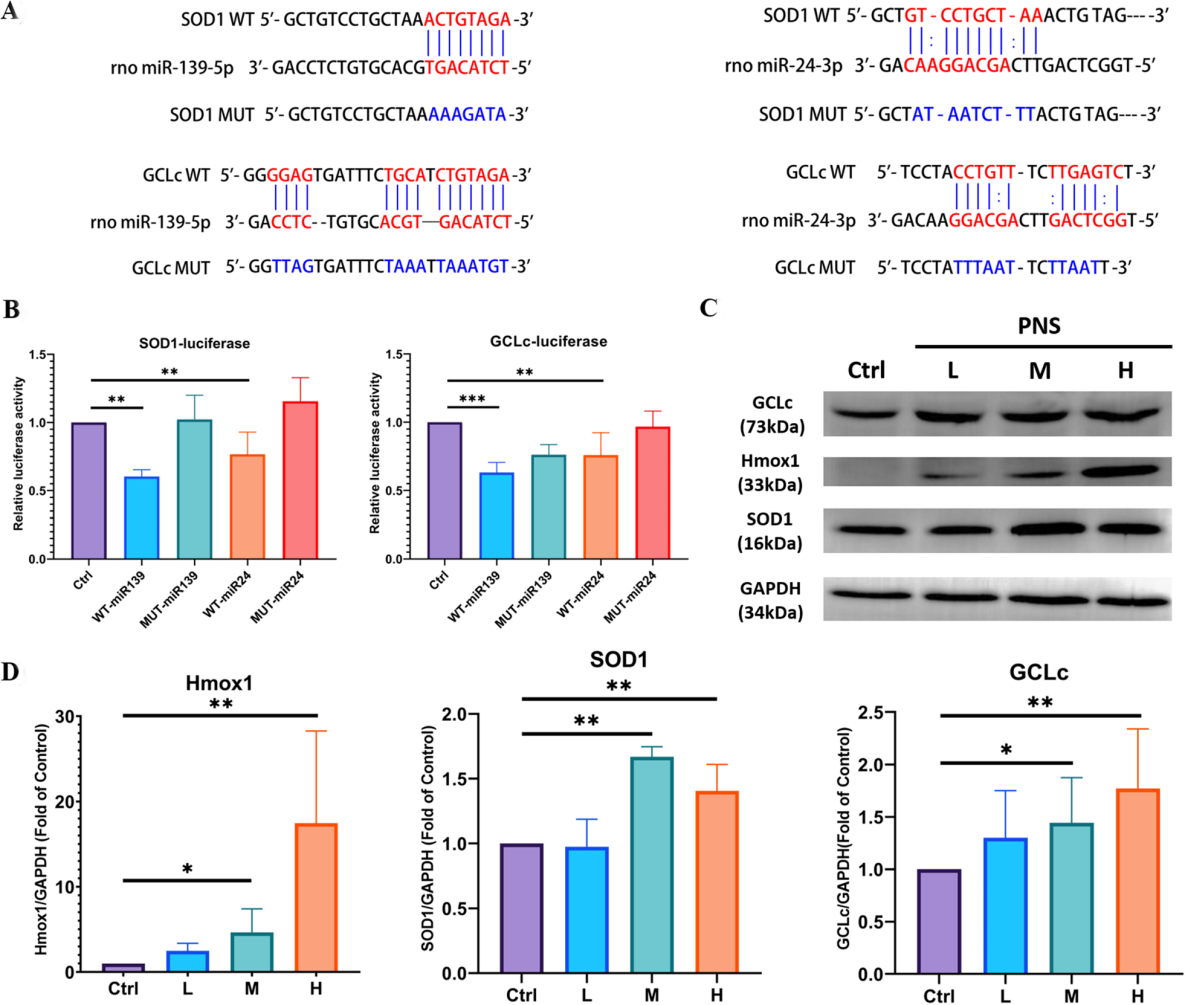
PNS regulates miR-139-5p and miR-24-3p to up-regulate ant-oxidation related proteins in INS-1 cells. **A** The binding sites of miR-139-5p and miR-24-3p on WT GCLc (red), and the mutant type GCLc (blue). **B** The relative luciferase activity with control or miR-139-5p mimics or miR-24-3p mimics overexpression in GCLc and SOD1 WT or MT groups *p < 0.05; **p < 0.01. **C** Representative immune blots **D** and quantification of GCLc, Hmox1, and SOD1 protein expression level with control or different PNS concentration treatment in INS-1 cells (L, 11.2 mg/mL; M, 20.2 mg/mL; and H, 29.1 mg/mL)

Quantitative real-time PCR (qPCR) was conducted to verify the expression profiles of these genes in RNA samples under three different concentrations of PNS. The results showed that most of the targets considered presented a consistent trend of differential gene expression induced by PNS with RNA-seq expression profiles (Fig. 6c, d). Moreover, western blot further confirmed total superoxide dismutase 1 (SOD1), heme oxygenase-1 (Hmox1) and glutamate cysteine ligase (GCLc) expression at protein level (Fig. 7c, d), supporting the hypothesis of PNS up-regulated antioxidants to ameliorate diabetes-induced oxidative stress.

Based on the reconstructed miRNA gene regulatory network (Fig. 5), we further examined their binding site at 3′UTR of four MTIs, miR-24-3p to GCLc and SOD1, and miR-139-5p to SOD1 and GCLc. Results suggested that miR-139-5p and miR-24-3p showed down-regulation post PNS treatment, while miR-671 expression appears unstable (Fig. 6e). Their target interactions from dual luciferase assay confirmed the four MTIs mentioned before, and miR-139-5p presented a higher affinity to both SOD1 and GCLc genes than miR-24-3p. Showcasing miR-139-5p as a more promising miRNA target (Fig. 7a, b). Functional enrichment analysis results of up- and down-regulated DEmiRs are shown in Additional file 18: Table S8.

**Fig. 8:**
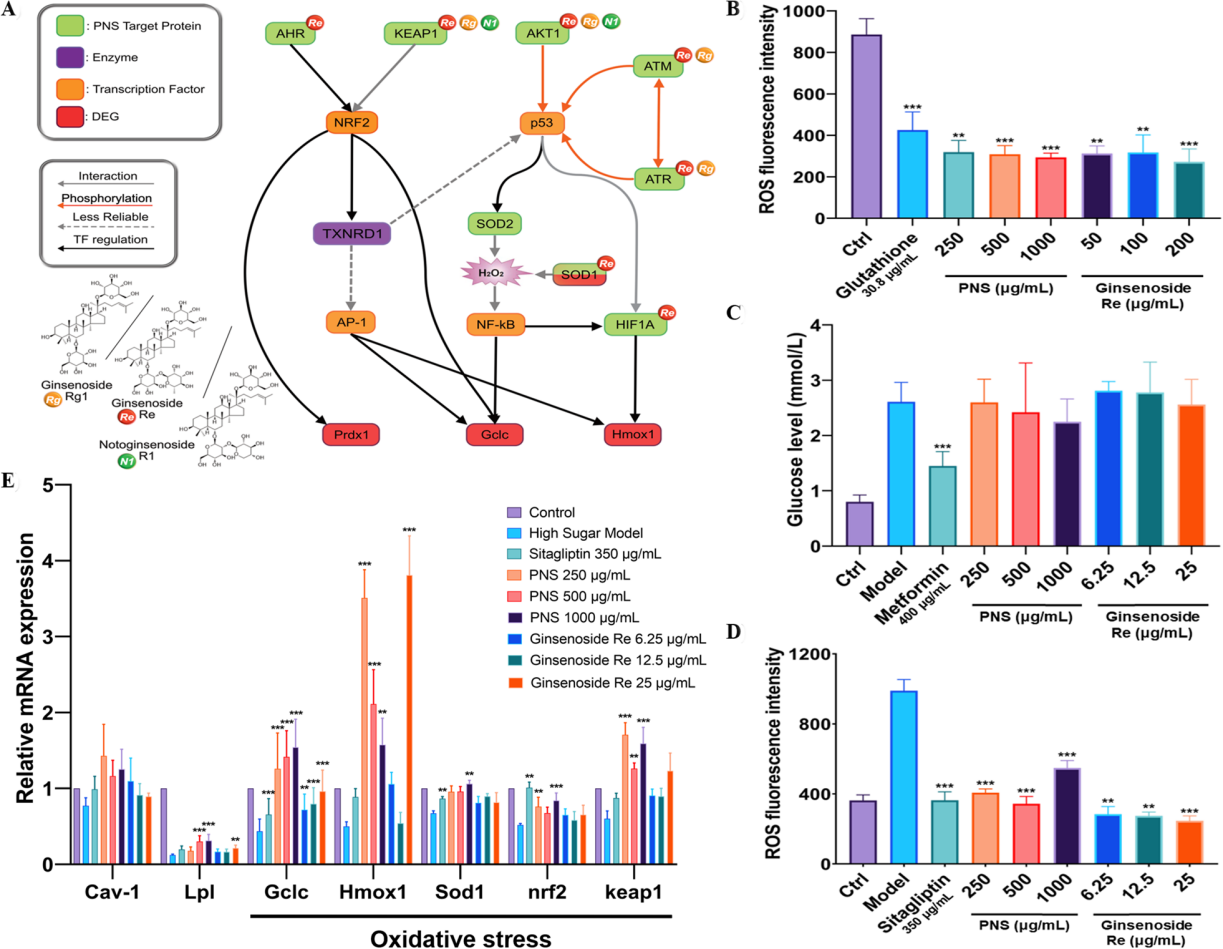
The effects of PNS on oxidative stress and glucose level in zebrafish. **A** PNS compound target proteins regulate anti-oxidants through TXNRD1 enzyme and transcription factors including NRF2, p53, AP-1 and NF-kB. Hmox1, SOD1, GCLc and PRDX1, takes place in the ROS suppression pathway and inhibit reactive oxygen radicals and hydrogen peroxide. Green rectangles are predicted PNS compound target proteins, purple rectangles are enzymes, orange rectangles are transcription factors and red rectangles are DEGs from our RNA-seq data. **B** Fluorescence value of ROS in WT zebrafish post PNS treatment. **C** Glucose and **D** ROS levels in high-sugar model zebrafish after PNS treatment. **E** Real-time qPCR results for selected genes involved in oxidative stress, insulin regulation and fatty acid metabolism. **p < 0.01; ***p < 0.001

We then drew a schematic diagram of how PNS compound targets can be linked to our DEGs shown in Fig. 8a. Interestingly, SOD1 is not only a DEG post PNS treatment, it is also a predicted target protein of PNS. Moreover, out of three major PNS components ginsenoside Rg1, Re and notoginsenoside R1, ginsenoside Re exhibited higher number of compound-target interactions, proving to have greater validation value, therefore was chosen for further exploratinon through in vivo validation along with PNS extract.

### Protective effects of PNS and ginsenoside Re in WT and high-glucose model Zebrafish

In vivo findings supported our previous results in rat pancreatic cells, both providing evidence for the hypothesis that PNS could reduce oxidative stress seen in metabolic syndromes. Results showed that both PNS and ginsenoside Re lowered ROS level in either WT or hyperglycemic model zebrafish (Fig. 8b, d), but showed little effect in glucose levels (Fig. 8c). The mRNA levels of genes related to anti-oxidation, inflammation, insulin regulation and fatty acid metabolism were determined, including SOD1, Hmox1, GCLc, Lpl, CAV-1, Keap1 and Nrf2. As shown in Fig. 8e, anti-oxidative genes were generally up-regulated under PNS treatment and exerted slightly stronger effects than single compound ginsenoside Re. A statistically significant up-regulation of GCLc and Hmox1 were observed in zebrafish treated with PNS (*p* < 0.01, or 0.001). Further details on in vivo experimental results are available in Additional file 25: Material and methods.

## Discussion

Recent attempts to uncover new therapeutic agents from natural derivatives to treat insulin resistance in diabetes and other metabolic disorders has been increasing. Chinese herbal medicine could be a plausible candidate. Transcriptomic data of drugs and diseases has been highly used to investigate treatments for the disease over the years and studies in various herbal compounds have provided the base with promising results. Their active compounds have lowered glucose and lipid levels with minimal side effects compared to modern medicine. PNS is commonly used in Asian countries and is one of the major species in Panax genus. It has potential therapeutic effects on many human chronic disorders including, arthritis, atherosclerosis, Alzheimer disease, hyperlipidemia and in our particular interest, oxidative stress and insulin resistance [39, 40]. By combining transcriptomic data with systems biology, we introduced the significantly affected mRNA and miRNA under PNS treatment and analyzed their cellular targets with associated signaling pathways. To the best of our knowledge, a handful of previous studies focused on mRNA and miRNA expression of PNS compounds on different disease models, but the evidence supporting the effect of PNS as a whole with the combination of experimental, transcriptomic data and network approach in the case of oxidative stress and insulin resistance is scant. Other disease models have shown PNS compounds with cholesterol reducing, oxidative stress lowering and cardio-cerebral vascular disease preventing properties [41, 42]. Many studies focused on bioactive compounds, such as Rb1, Rg3, and Rb2, where Rb1 regulates glycolipid metabolism and improves insulin and leptin sensitivities [11], and Rg3 increases insulin secretion of INS-1 cells in high-glucose environment [12]. However, compound-level studies can’t represent the complex interaction networks of PNS function, so our study takes into account the entirety of the herbal plant to map the multi-component and multi-channel mechanism.

The detailed understanding of the molecular mechanism remains unclear, but proves crucial for improvement of disease treatment. In the present study, we questioned whether PNS has direct beneficial effects on insulin resistance and determined the relevant mRNA and miRNA expression profiles in PNS supplemented pancreatic β cell samples compared with control samples based on NGS. We extracted metabolic disorder related genes from the RNA-seq matrix. Results presented over half of extracted genes with a positive correlation to PNS dosage effect on gene expression compared to the control model (Additional file 5: Fig. S5). We then generated the biological functions, canonical pathways and miRNA-mRNA networks related to our list of DEGs. We found that functional classification showed that significantly up- and down-regulated genes are related to three significant mechanisms, insulin regulation, reactive oxygen species (ROS) response and fatty acid metabolism (Fig. 2). Under PNS treatment, insulin regulation and lipid metabolism were mainly related to DEG down-regulation, while reduced ROS stress correlated with DEG up-regulation (Fig. 4).

### PNS exposure alleviates oxidative stress response

**Fig. 9:**
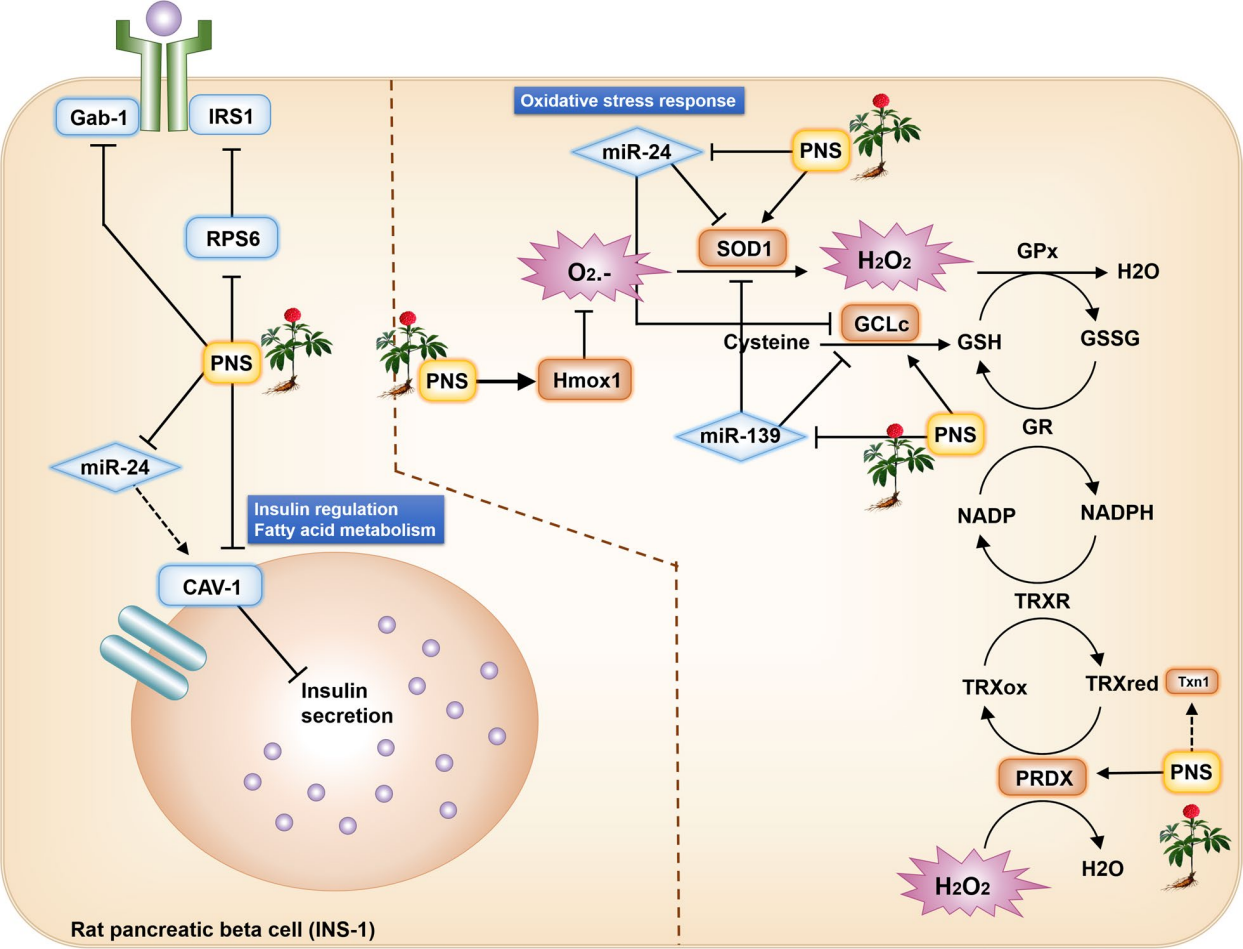
PNS induced β cell autocrine signaling response. PNS regulates different genes and proteins at multiple levels. Following the uptake of PNS, it up-regulates gene expression of Hmox1, SOD1, GCLc, Txn1 and PRDX (arrows) while suppressing Gab-1 and CAV-1 gene. Hmox1, SOD1, GCLc, Txn1 and PRDX, takes place in the ROS suppression pathway and inhibit reactive oxygen radicals and hydrogen peroxide. Blue rectangles and rhombus are suppressed DEGs and DEmiRs, respectively and orange rectangles are up-regulated DEGs. Hmox1, heme oxygenase-1. SOD1, superoxide dismutase 1. GCLc, glutamate cysteine ligase. Txn1, thioredoxin. PRDX, peroxiredoxin. Gab-1, GRB2 associated binding protein 1. RPS6, ribosomal protein S6 kinase. CAV-1, caveolin-1

Elevated levels of pro-oxidants and lower levels of antioxidants such as glutathione are primarily seen in patients with metabolic disorders such as diabetes. It is increasingly understood that glucose metabolism is regulated by redox homeostasis, where the redox imbalance contributes to insulin resistance and hyperglycemia development [43, 44]. In this study, we found that, through pathway crosstalk analysis, PNS treatment suppressed oxidative stress factors, by up-regulating antioxidant-related gene expression levels, including superoxide dismutase 1 (SOD1), peroxiredoxin 1 (PRDX1), heme oxygenase-1 (Hmox1) and glutamate cysteine ligase (GCLc), indicating PNS could reduce insulin resistance. However, ROS may vary in reactivity and molecular targets. Therefore, mapping ROS signals’ biological effects prove challenging to decode. Decreased glutathione (GSH) content in cells is commonly found in human diabetes, accompanied by increased oxidative stress and the induction of the GSH redox system [44, 45]. GSH redox system is an antioxidant response, shown to have beneficial effects on insulin sensitivity, but the mechanistic link remains incompletely understood. Our findings reveal PNS provision up-regulated GCLc gene expression. GCLc is the rate limiting enzyme in GSH synthesis that directly repress ROS [46, 47]. Interestingly, the down-regulation of miR-139-5p and miR-24-3p under PNS exposure both positively affect GCLc expression as GCLc targets is a target of both miRNAs (Fig. 9). In other words, PNS suppresses the interference of miR-139-5p and miR-24-3p on GCLc function, while inducing GCLc expression, further ensuring the proper transformation of cysteine to GSH.

Deficiencies in antioxidant defenses against ROS described in diabetes included antioxidant enzyme such as SOD1 [48, 49], PRDX1 [50], Hmox1 [51] and antioxidants, thioredoxin 1 (Txn1) [52]. SOD1 encodes for copper zinc superoxide dismutase 1, this enzyme is mainly found in the cytosol and directly converts superoxide to hydrogen peroxide (H_2_O_2_) and oxygen (O_2_). Deletion of this enzyme results in glucose intolerance [53]. SOD1, GCLc, PRDX1, Txn1 are all related in the endogenous defense system, where SOD1 eliminates O_2_.-, whereas PRDX1, GCLc, and Txn1 involve in the elimination of H_2_O_2_ [54]. Therefore, we propose PNS exposure to rat pancreatic β cells positively affects insulin resistance by up-regulating the expression of antioxidant defense-related genes (Fig. 9). In our study, miR-24-3p and miR-139-5p appear to suppress the expression of SOD1 and GCLc, whilst PNS down-regulated both miRNAs. As a result, PNS suppresses the interference of miR-24-3p and miR-139-5p on SOD1 and GCLc function. With these two positively regulated loops working together, causing an enhanced oxidant reducing function.

### PNS exposure reduces insulin regulation and lipid imbalance

In gene-pathway network, two down-regulated DEGs were notable, first being the loss of ATP binding cassette subfamily G member 1 (Abcg1), which functions in removing excess cholesterol, impaired insulin secretion both in vivo and in vitro [55]. Second, both overexpression [56, 57] and loss [58–60] of lipoprotein lipase (Lpl) impairs insulin secretion as Lpl serves as a “gatekeeper”. ETS variant transcription factor 5 (Etv5) gene, another major regulator of insulin secretion, is significantly enriched in human diabetes and obesity GWAS [61], and in our case, was one of the central hub genes in the up-regulated DEGs related MTI network shown in Fig. 5. MiR-24-3p/198b/671 and let-7f all targeted Etv5, but let-7f has been seen to induce instead of suppress Etv5 expression. MiR-24-3p and miR-671 both negatively influenced Etv5, suggesting the down-regulation of miR-24-3p and miR-671 under PNS treatment were involved in protection against cytotoxic ROS (presented by reduced inhibition of GCLc and PRDX1) as well as lowered inhibition effect on insulin secretion [46, 62, 63].

PNS treatment down-regulated the expression of miR-24-3p, which induced the expression of caveolin-1 (CAV-1). CAV-1, plays a role in insulin-receptor mediated signaling, where siRNA knockdown resulted in a significant increase in insulin secretion in several pancreatic cell lines, including INS-1 and MIN6 [64, 65]. GRB2-associated binding protein 1 (Gab-1), is an insulin receptor substrate that recruits downstream signaling elements that may be part of signaling pathways leading to cell growth, transformation, and apoptosis [66]. The downstream effect of Gab-1 gene suppression under PNS treatment is unclear. Our enrichment results didn’t show any further DEGs related to this gene and none of the DEmiRs regulated Gab-1.

Lpl is an enzyme that hydrolyzes triglycerides into fatty acids. Insulin stimulates Lpl expression and Lpl activity is lower in patients with diabetes, which in turn impair lipoproteins’ metabolism, leading to hyperglyceridemia [67]. Transgenic mice with liver-specific overexpression of Lpl develop liver-specific lipid accumulation and liver-specific insulin resistance [56], and patients with conditions that results in Lpl mutation (hyperlipoproteinemia type 1) that lower Lpl expression is prone to developing insulin resistance, not necessarily specific to the liver [57]. In our study, PNS appears to suppress Lpl. However, further research must be done to decipher the exact mechanism of action in regulating lipid imbalance.

### An unconventional approach to deciphering protective functions of PNS

Multiple compounds characterize a single TCM, in our case, *Panax notoginseng* consists of over one hundred compounds, details available in Additional file 20: Table S10. Through, UPLC-MS/MS analysis of the PNS sample used in this study, we identified nine major components of PNS including notoginsenoside R1, ginsenoside Rb1, Rb2, Rb3, Rd, Re, Rg1, Rg2 and Rg3, marked on the based peak chromatogram of positive ion mode shown in Additional file 8: Fig. S8. Detailed data results and linearity plot of nine reference standards for quantitation are listed in Additional file 19: Table S9, Additional files 9 and 10: Figs. S9 and S10. Our study aims to evaluate the mechanism of PNS through an unconventional method based on the mixture of compounds instead of single compounds and results demonstrated that our PNS sample identified active compounds well known in PNS research and identified anti-oxidative mechanism as a main target explaining the protective effect of PNS when treating metabolism disorders such as insulin resistance as oxidative stress is a well-known cause of insulin resistance. Anti-oxidative functions were also validated in animal model. With these supporting results, we reclaim that we are trying to elucidate the whole system not through a conventional means of research using single compounds. Instead, the use of a systems biology-based approach based on transcriptome profiling with experimentally verified targets of these PNS active compounds, we can therefore figure out a mapping of how different compounds link to alleviating oxidative stress.

## Conclusion

In summary, our research into the protective functions of PNS shows its’ mechanisms through multiple targets, biological processes and signaling pathways especially involving the regulation of anti-oxidation related genes and miRNAs. Overall, this study contributes to the investigation of an alternative approach in explaining anti-oxidative protecting mechanisms of medicinal plants based on systems biology and transcriptome profiling where the combination of omics data with systems biology strategy could aid in the exploration of potential functions and molecular mechanisms of Chinese herbal medicine.

## Supplementary Information

**Additional file 1. Fig. S1**: Read quality of RNA-seq and miRNA-seq data. **A** PCA for pre-processed mRNA-seq data; **B** normalized mRNA-seq data. High: 29.1 mg/mL, Medium: 20.2 mg/mL; and Low: 11.2 mg/mL.

**Additional file 2. Fig. S2**: Volcano plot of DEGs in **A** low to control; **B** medium to control; **C** high to control.

**Additional file 3. Fig. S3**: Volcano plot of DEmiRs in **A** low PNS to control; **B** medium PNS to control; **C** high PNS to control; **D** medium PNS to low PNS and **E** high PNS to medium PNS. High: 29.1 mg/mL, Medium: 20.2 mg/mL; and Low: 11.2 mg/mL.

**Additional file 4. Fig. S4**: Overlapping DEGsin L/M/H to control in **A** up regulated DEGs; **B** down regulated DEGs.

**Additional file 5. Fig. S5**: Insulin-related genes from the entire RNA-seq matrix with the heat map showing over half of extracted genes had a positive correlation to increased and decreased PNS dosage on gene expression in INS-1 compared to control model.

**Additional file 6. Fig. S6**: Functional enriched clustering annotation on **A** up regulated MTI related DEGs; and **B** down regulated MTI related DEGs.

**Additional file 7. Fig. S7**: Confirmed up and down regulated DEmiRs related gene network from miRTarBase with experimental support. The grey rectangles denote genes with experimental support, the rhombus represents our DEmiRs, red denoting up-regulated and blue as down-regulated.

**Additional file 8. Fig. S8**: Total ion chromatogramof UPLC–MS/MS analysis of PNS under positive ion mode.

**Additional file 9. Fig. S9**: UPLC-MS/MS spectra of main components in *Panax notoginseng*and 9 reference compounds of PNS. 1: Notoginsenoside R1, 2: Ginsenoside Re, 3: Ginsenoside Rg1, 4: Ginsenoside Rb1, 5: Ginsenoside Rg2, 6/7: Ginsenoside Rb2/Rb3, 8: Ginsenoside Rd and 9: Ginsenoside Rg3.

**Additional file 10. Fig. S10**: Linearity plot for 8 standard solutions over a range of 0.5–500 ng/µL. PNS reference standards including notoginsenoside R1, ginsenoside Rb1, Rb2, Rb3, Rd, Re, Rg1, Rg2 and Rg3.

**Additional file 11. Table S1**: mRNA raw data from RNA sequencing post PNS treatment.

**Additional file 12. Table S2**: mRNA CPM matrix after normalization and pretreatment.

**Additional file 13. Table S3**: miRNA raw data from miRNA sequencing post PNS treatment.

**Additional file 14. Table S4**: Up- and down-regulated DEGs under three concentrations of PNS.

**Additional file 15. Table S5**: Up- and down-regulated DEmiR under three concentrations of PNS.

**Additional file 16. Table S6**: Up-regulated DEGs functional enrichment analysis results.

**Additional file 17. Table S7**: Down-regulated DEGs functional enrichment analysis results.

**Additional file 18. Table S8**: Up- and down-regulated DEmiRs functional enrichment analysis results.

**Additional file 19. Table S9**: PNS metabolite UPLC–MS/MS analysis report.

**Additional file 20. Table S10**: Known PNS compounds derived from HERB database.

**Additional file 21. Table S11**: PNS metabolite target protein prediction with DeepConv-DTI, SEA, and SuperPred.

**Additional file 22. Table S12**: Functional roles and interactions of PNS target proteins to DEGs.

**Additional file 23. Table S13**: Experimental dose of PNS and ginsenoside Re for in vivo evaluation.

**Additional file 24. Table S14**: Primers used in zebrafish model for gene expression analysis.

**Additional file 25.** Material and methods.

## Data Availability

Not applicable.

## Abbreviations

NGS: Next-generation sequencing
DEGs: Differentially expressed genes
DEmiRs: Differentially expressed miRNAs
FDR: False discovery rate
TCMs: Traditional Chinese Medicines
PNS: *Panax notoginseng*
SQ: Sanqi
NR1: Notoginsenoside R1
INS-1: Rat pancreatic β cells
MTIs: MiRNA target interactions
RT-qPCR: Real time-quantitative PCR
SOD1: Superoxide dismutase 1
Hmox1: Heme oxygenase-1
GCLc: Glutamate cysteine ligase
PRDX1: Peroxiredoxin 1
GAPDH: Glyceraldehyde 3-phosphate dehydrogenase
Txn1: Thioredoxin 1
GSH: Glutathione
ROS: Reactive oxygen species
Abcg1: ATP binding cassette subfamily G member 1
Lpl: Lipoprotein lipase (Lpl)
Etv5: ETS variant transcription factor 5
CAV-1: Caveolin-1
Gab-1: GRB2-associated binding protein 1
