# Supplementary figures and images for: Panax notoginseng alleviates oxidative stress through miRNA regulations based on systems biology approach
Source: Chin Med. 2023 Jun 20;18:74. doi: 10.1186/s13020-023-00768-y (PMC10280844; doi:10.1186/s13020-023-00768-y)

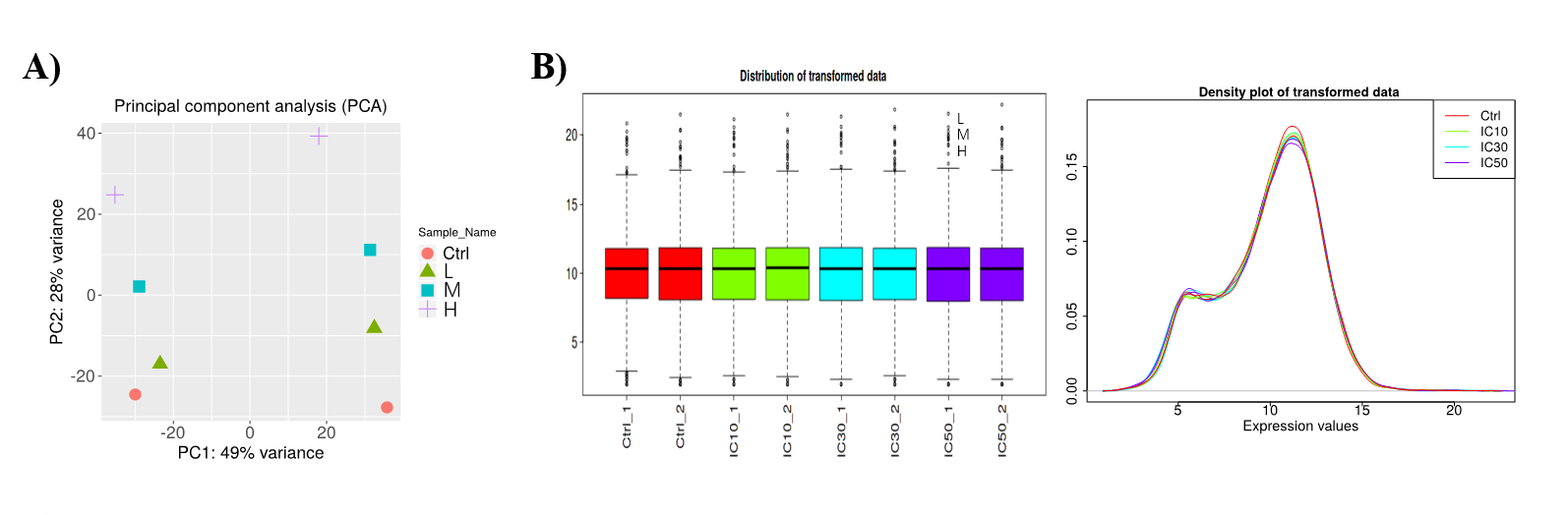

Supplement: Supplementary file 1 — Additional file 1. Fig. S1: Read quality of RNA-seq and miRNA-seq data. A PCA for pre-processed mRNA-seq data; B normalized mRNA-seq data. High: 29.1 mg/mL, Medium: 20.2 mg/mL; and Low: 11.2 mg/mL. [file 13020_2023_768_MOESM1_ESM.png]

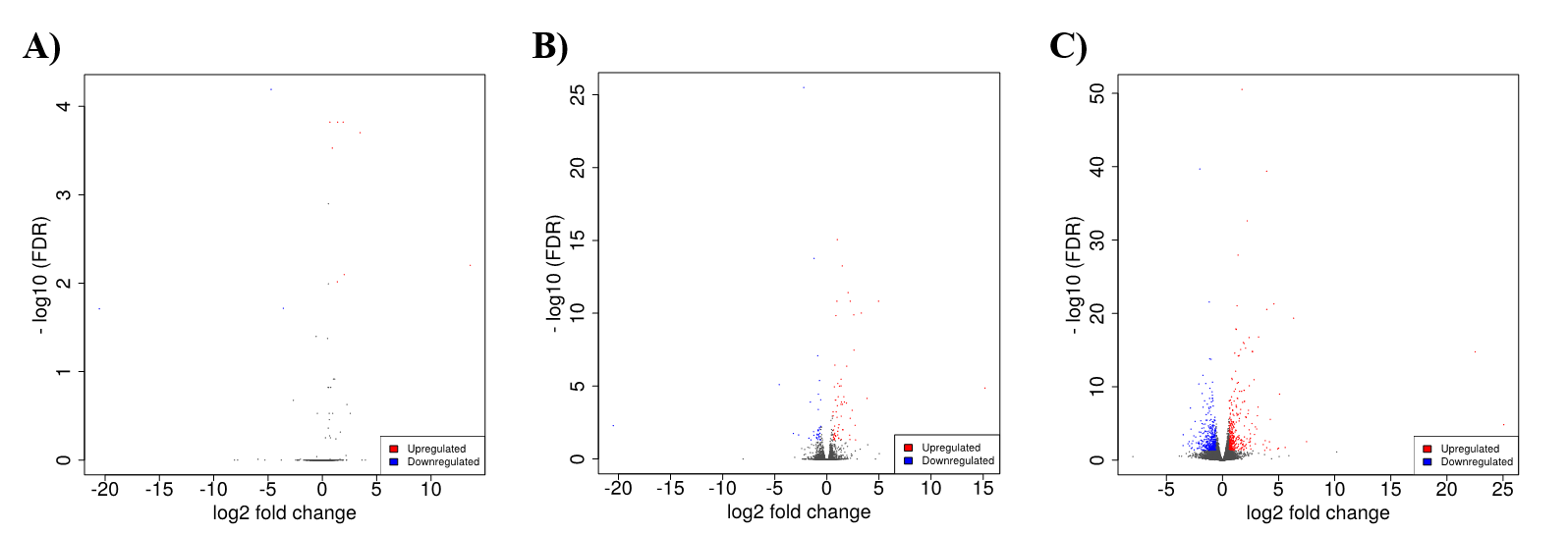

Supplement: Supplementary file 2 — Additional file 2. Fig. S2: Volcano plot of DEGs in A low to control; B medium to control; C high to control. [file 13020_2023_768_MOESM2_ESM.png]

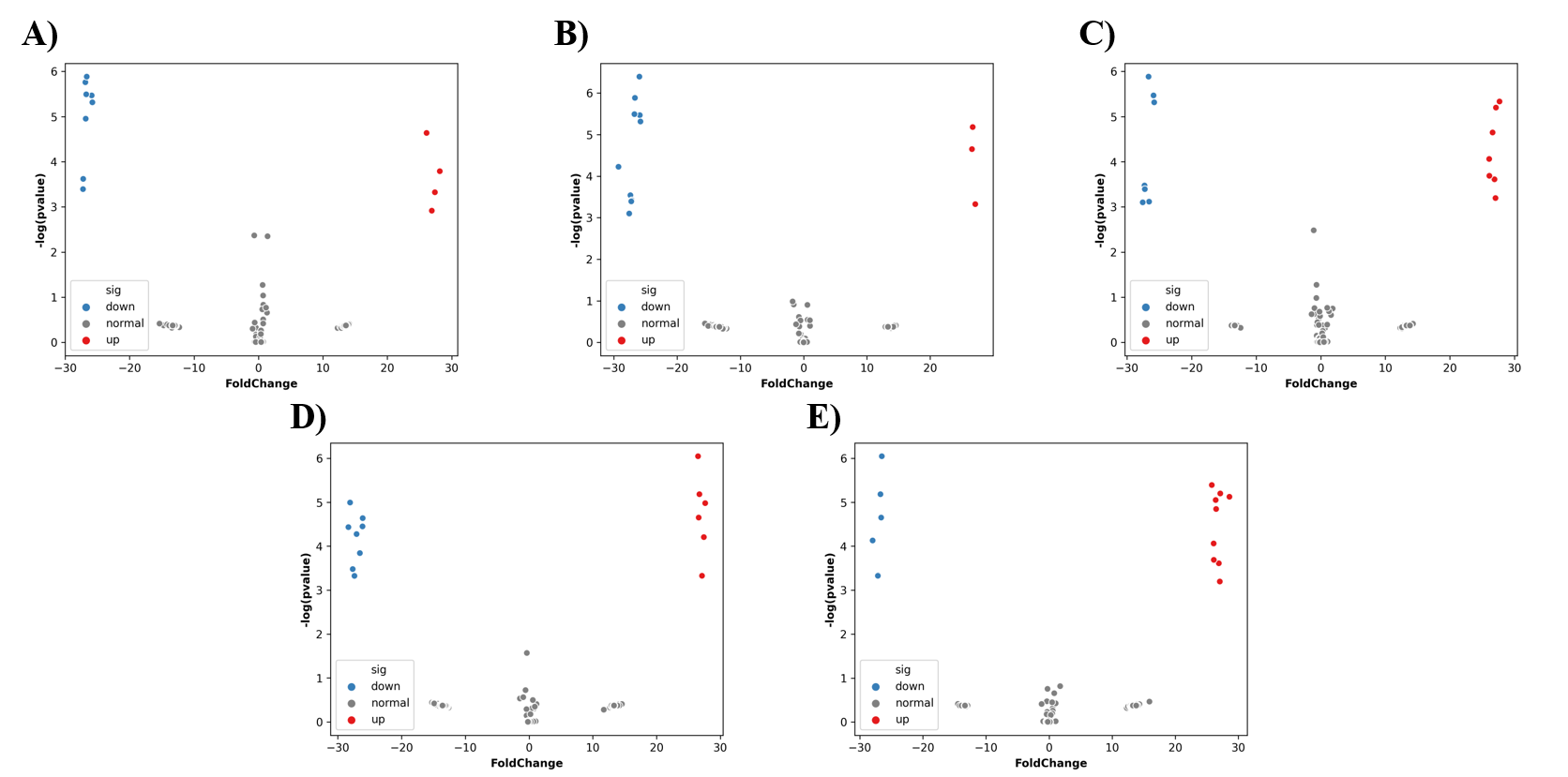

Supplement: Supplementary file 3 — Additional file 3. Fig. S3: Volcano plot of DEmiRs in A low PNS to control; B medium PNS to control; C high PNS to control; D medium PNS to low PNS and E high PNS to medium PNS. High: 29.1 mg/mL, Medium: 20.2 mg/mL; and Low: 11.2 mg/mL. [file 13020_2023_768_MOESM3_ESM.png]

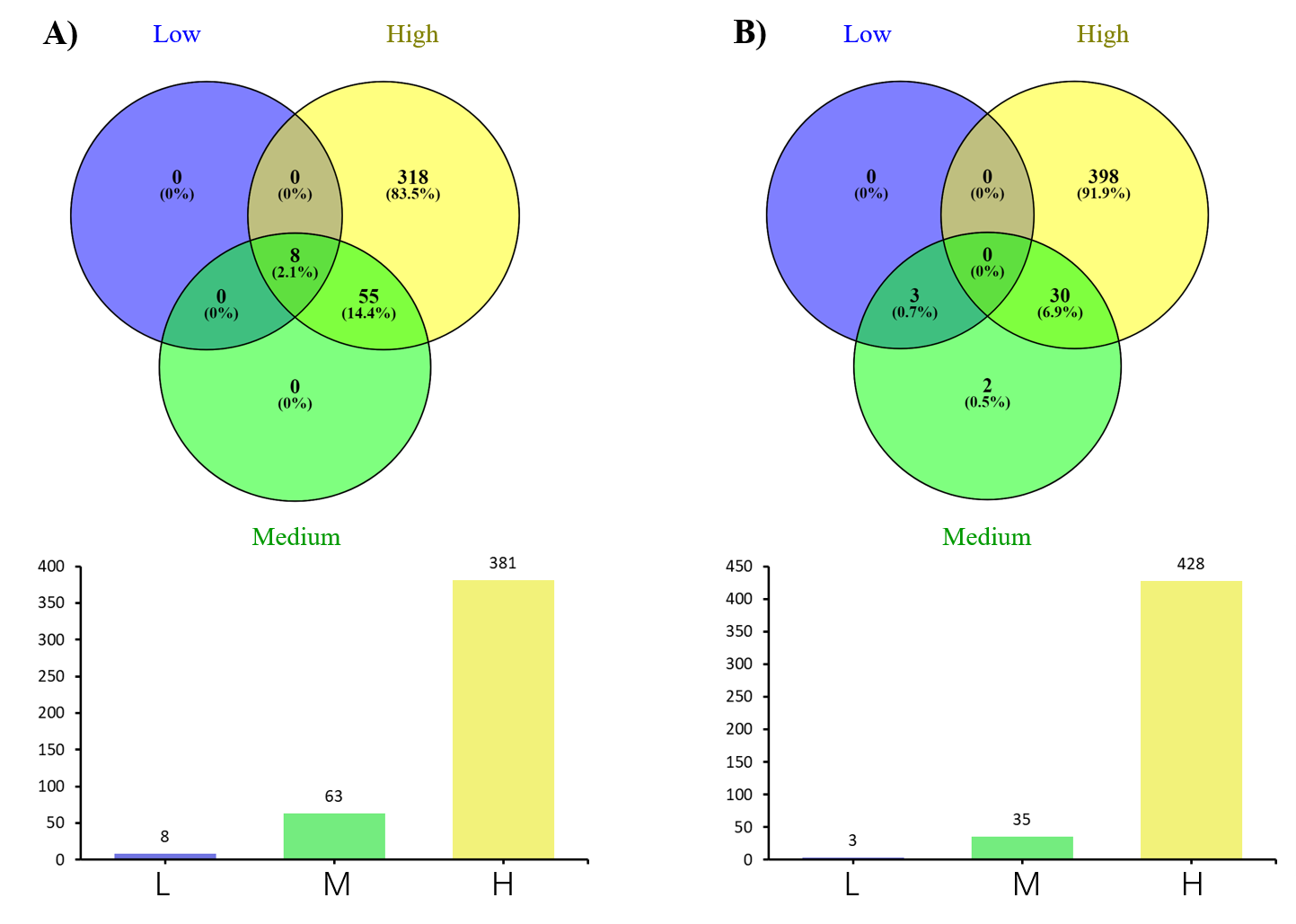

Supplement: Supplementary file 4 — Additional file 4. Fig. S4: Overlapping DEGsin L/M/H to control in A up regulated DEGs; B down regulated DEGs. [file 13020_2023_768_MOESM4_ESM.png]

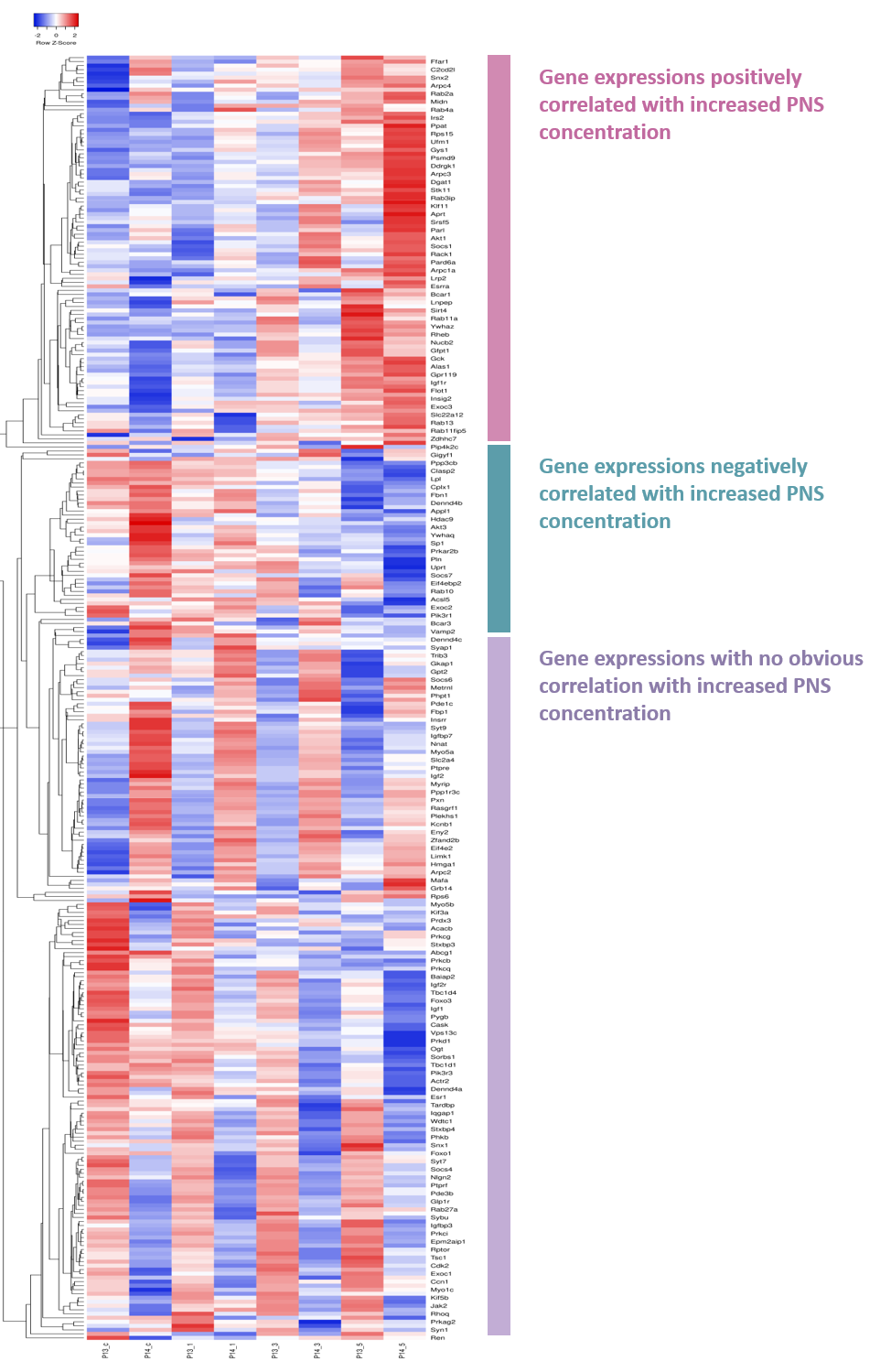

Supplement: Supplementary file 5 — Additional file 5. Fig. S5: Insulin-related genes from the entire RNA-seq matrix with the heat map showing over half of extracted genes had a positive correlation to increased and decreased PNS dosage on gene expression in INS-1 compared to control model. [file 13020_2023_768_MOESM5_ESM.png]

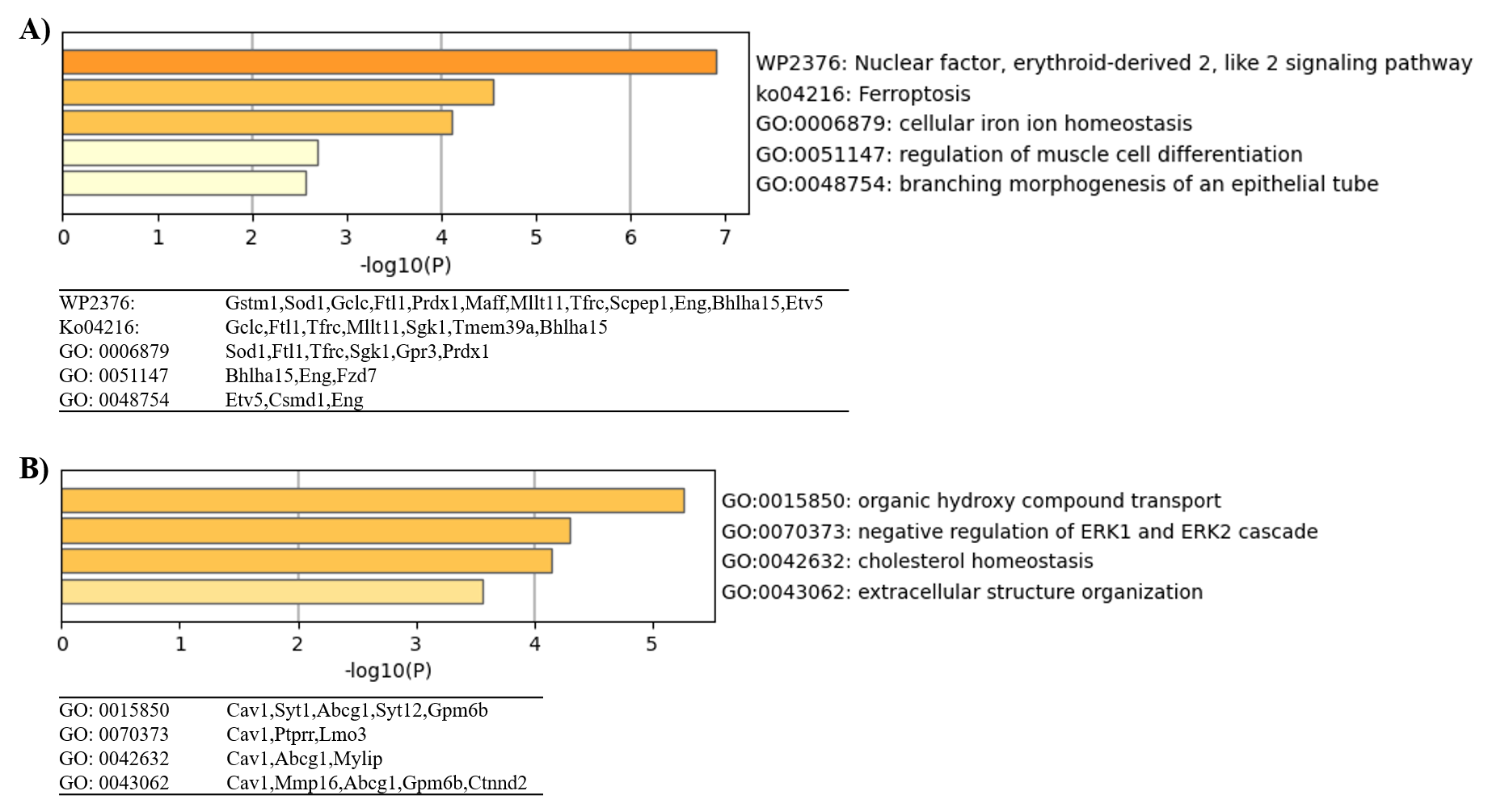

Supplement: Supplementary file 6 — Additional file 6. Fig. S6: Functional enriched clustering annotation on A up regulated MTI related DEGs; and B down regulated MTI related DEGs. [file 13020_2023_768_MOESM6_ESM.png]

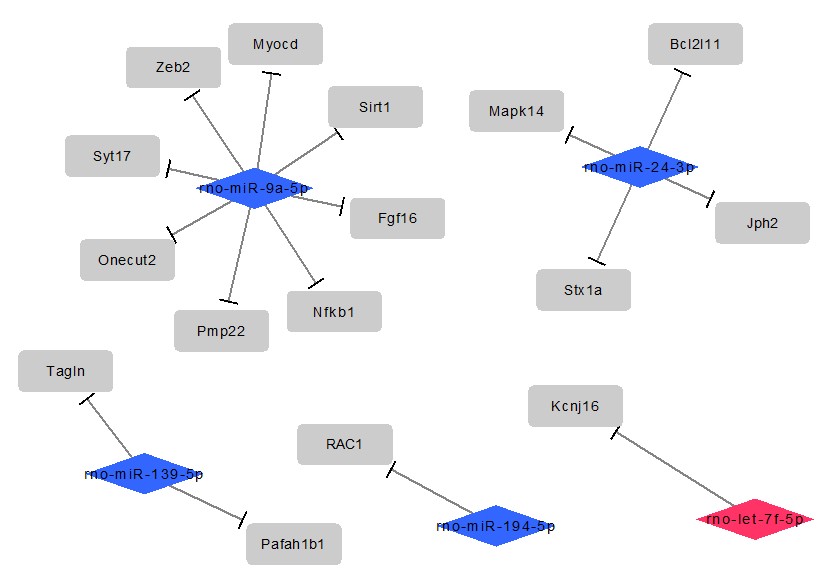

Supplement: Supplementary file 7 — Additional file 7. Fig. S7: Confirmed up and down regulated DEmiRs related gene network from miRTarBase with experimental support. The grey rectangles denote genes with experimental support, the rhombus represents our DEmiRs, red denoting up-regulated and blue as down-regulated. [file 13020_2023_768_MOESM7_ESM.png]

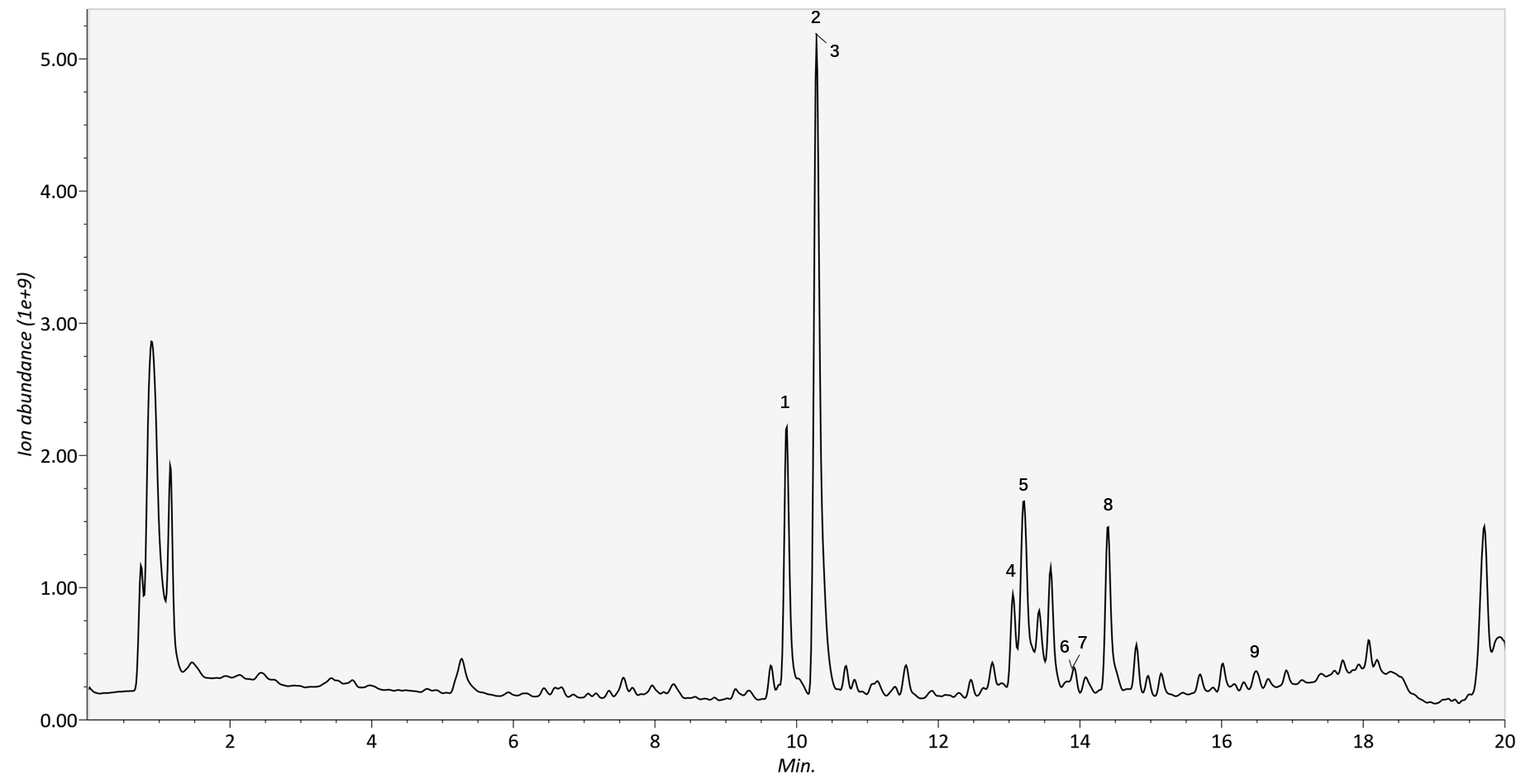

Supplement: Supplementary file 8 — Additional file 8. Fig. S8: Total ion chromatogramof UPLC–MS/MS analysis of PNS under positive ion mode. [file 13020_2023_768_MOESM8_ESM.png]

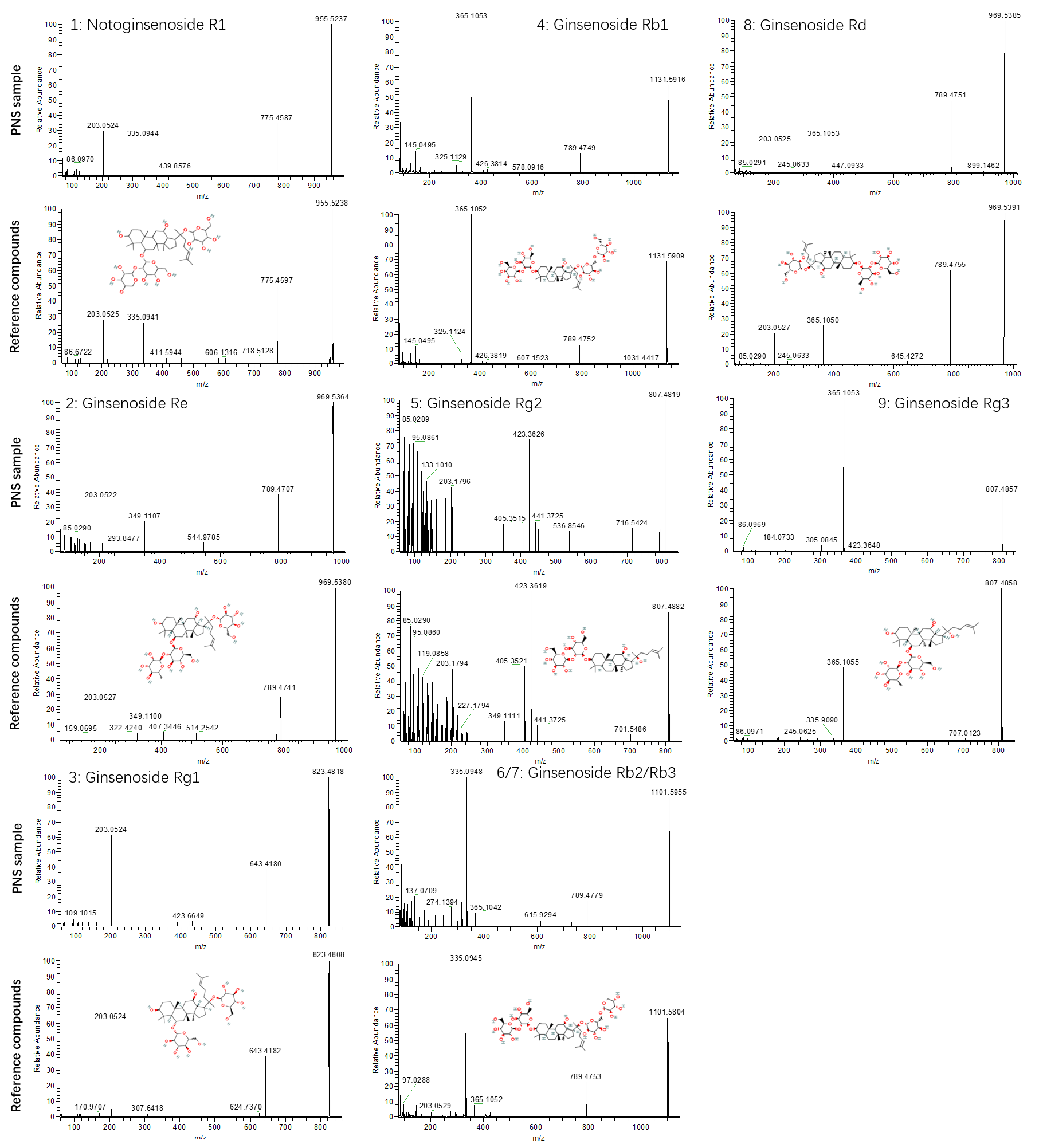

Supplement: Supplementary file 9 — Additional file 9. Fig. S9: UPLC-MS/MS spectra of main components in Panax notoginsengand 9 reference compounds of PNS. 1: Notoginsenoside R1, 2: Ginsenoside Re, 3: Ginsenoside Rg1, 4: Ginsenoside Rb1, 5: Ginsenoside Rg2, 6/7: Ginsenoside Rb2/Rb3, 8: Ginsenoside Rd and 9: Ginsenoside Rg3. [file 13020_2023_768_MOESM9_ESM.png]

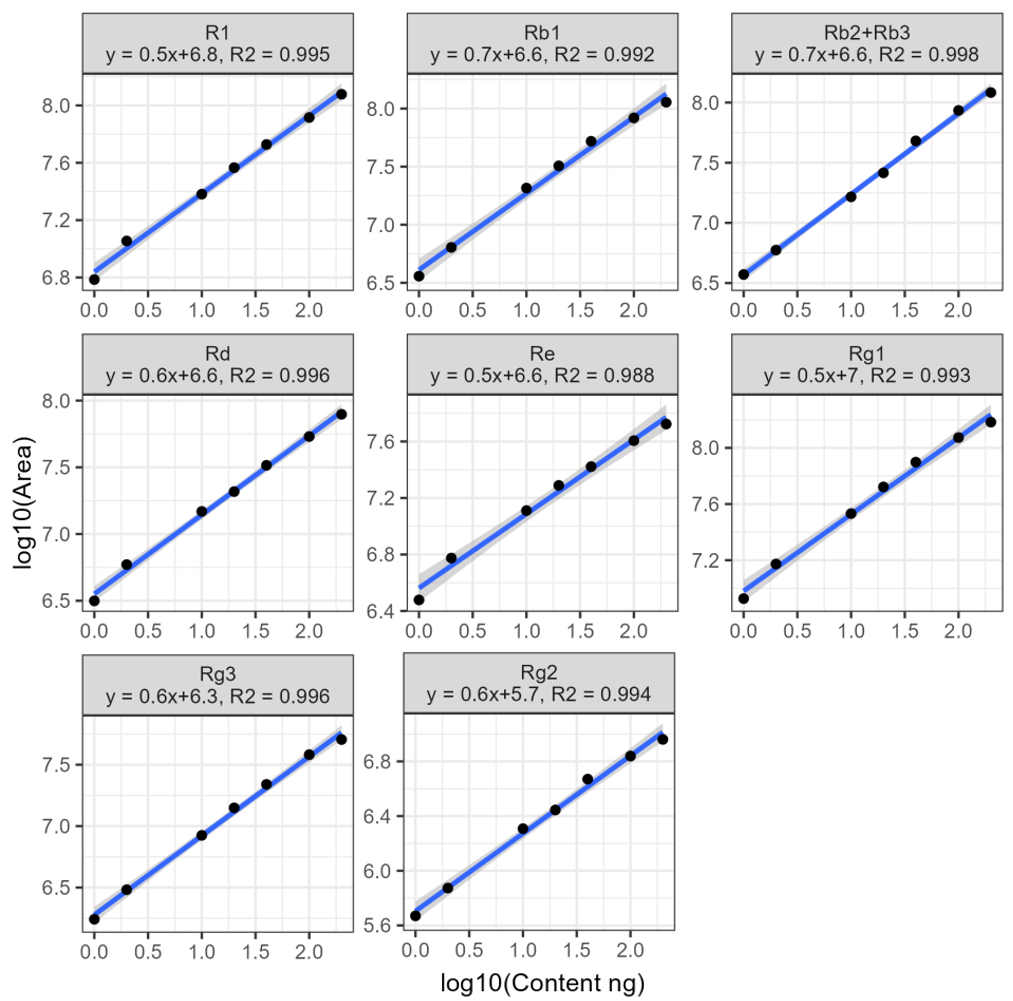

Supplement: Supplementary file 10 — Additional file 10. Fig. S10: Linearity plot for 8 standard solutions over a range of 0.5–500 ng/µL. PNS reference standards including notoginsenoside R1, ginsenoside Rb1, Rb2, Rb3, Rd, Re, Rg1, Rg2 and Rg3. [file 13020_2023_768_MOESM10_ESM.png]
