# Supplementary material for: Panax notoginseng alleviates oxidative stress through miRNA regulations based on systems biology approach
Source: Chin Med. 2023 Jun 20;18:74. doi: 10.1186/s13020-023-00768-y (PMC10280844; doi:10.1186/s13020-023-00768-y)
Supplement: Supplementary file 25 — Additional file 25. Material and methods. [file 13020_2023_768_MOESM25_ESM.docx]

**Material and Methods**

Preparation of calibration curve and quality control samples

Mixed standard stock solution containing notoginsenoside R1, ginsenoside Rb1, ginsenoside Rb2, ginsenoside Rd, ginsenoside Re, ginsenoside Rg1, ginsenoside Rg2, ginsenoside Rg3 and ginsenoside Rh1 at concentrations 500 ng/μL was prepared by dissolving the accurately weighed reference compounds in 50% acetonitrile, and further diluted to a series of concentrations ranging from 0.5 ng/μL to 500 ng/μL.

Mass Spectrometry of PNS reference compounds

Qualitative and quantitative analysis of nine major components of PNS were performed using Ultimate 3000 UPLC with BEH C18 column (2.1 x 100 mm, 1.7 µm) following Q-Exactive mass spectrometer. Parallel reaction monitoring (PRM) mode was used to acquire the targeted data. Positive mode was used to detect the 9 selected compounds in a single run. Linearity plot in Additional File Figure S10 was used for quantitative analysis and the content of 9 selected compounds in PNS sample are shown in Additional File Table S9. Data were processed using the XcaliburTM 4.1 software (ThermoFisher, San Jose, CA, USA, 2019) and Skyline software [1].

Experimental animals

Two types of zebrafish were used in this study: wild-type AB strain zebrafish and high-sugar and high-fat induced. Adult zebrafish were housed in light- and temperature-controlled aquaculture facility with a standard 14:10 h light/dark photoperiod and fed with live brine shrimp twice daily and dry flake once a day. Reproduction of embryos was conducted in a 2:1 ratio, female to male respectively. On average, 200-300 embryos were generated. Embryos were maintained at 28 °C fish water containing 0.2% Instant Ocean Salt in deionized water, in pH 6.9-7.2, an electrical conductivity of 480-510 μS/cm and hardness of 53.7-71.6 mg/L CaCO_3_. Egg yolk powder (20200809, Zhejiang Ags Biotech Co., Ltd., China) and glucose (I2209335, Shanghai Aladdin Biochemical Technology Co., Ltd., China) were co-fed to zebrafish to induce a high-sugar and high-fat model. 60% of egg yolk powder is lipid, and feeding zebrafish with glucose can rapidly increase the glucose content in blood of zebrafish. Sustained high blood sugar levels mimic diabetic complications.

Maximum Tolerable Concentration (MTC) determination in zebrafish

To determine the maximum tolerated concentration (MTC) of PNS and ginsenoside Re in two different models, two days-post-fertilization (dpf) zebrafish were randomly distributed into 6-well plates, with 30 larvae in 3 mL of fish water in each well and mortality was recorded at post 24 h treatment. In the TCM treatment groups, a range of PNS extract concentrations (125, 250, 500, 1000 and 2000 μg/mL) or ginsenoside Re (12.5, 25, 50, 100 and 200 μg/mL) was added to wells and incubated for 24 h.

Reactive Oxygen Species (ROS) Assay

There are oxygen free radicals (ROS) in the zebrafish body, and CM-H_2_DCFDA (General Oxidative Stress Indicator, C6827, Invitrogen, USA) fluorescent probe from ROS assay kit (NanjingJiancheng Bioengineering Institute, Nanjing, China) was used to react with the ROS in zebrafish, and the fluorescence value after the reaction is detected. CM-H_2_DCFDA is hydrolyzed into DCFH, DCFH is a polar chemical substance that remains in the cell. High fluorescence intensity DCF is produced when the oxidant reacts with DCFH.

3 dpf (days post fertilization) wild-type zebrafish of AB strain were randomly selected and placed in a 6-well plate. 30 zebrafish were treated in each well (experimental group). PNS sample and positive control glutathione (CAS:70-18-8, Sigma, USA) concentration of 30.8 μg/mL or 350 μg/mL sitagliptin (H008754, Merck Sharp& Dohme Italia SPA) were administered into the water solution. The volume was fixed with ROS fluorescence detection solution, and the volume of each well was 3 mL. Zebrafish from each experimental group were then transferred into a 96-well plate, 2 fish/100 μL/well and ROS was measure at 488nm under a multimode microplate reader (SPARK, TECAN, Switzerland). Efficacy was evaluated after 2 days of treatment at 28 °C.

Biochemical Assay in High glucose zebrafish model

5 dpf (days post fertilization) wild-type zebrafish of AB strain were randomly selected in beakers, and 30 zebrafish were treated in each beaker (experimental group). The PNS samples were dissolved in water, the positive control of glucose assay was metformin (ACD0775, Sino-American Shanghai Squibb Pharmaceuticals Ltd., China) at a concentration of 400 μg/mL, and the positive control of ROS assay sitagliptin phosphate concentration was 350 μg/mL. At the same time, a normal control group and a model control group were set up. The volume of each cup was 25 mL. Except for the normal control group, all other experimental groups were fed with high-sugar and high-fat diet in water solution to establish the hyperglycemia model of zebrafish. Efficacy evaluation was performed after 2 days of treatment at 28°C. Glucose levels were measured using blood glucose meter (ACCU-CHEK Performa, Roche Diagnostic Products (Shanghai) Co., Ltd., China).

Gene expression analysis in High glucose zebrafish model

To further investigate the potential antioxidative mechanism of PNS and ginsenoside Re, superoxide dismutase 1 (SOD1), heme oxygenase-1 (Hmox1), glutamate cysteine ligase (GCLc), lipoprotein lipase (Lpl), caveolin-1 (CAV-1), Kelch-like ECH-associated protein 1 (Keap1) and nuclear factor erythroid 2–related factor 2 (Nrf2) were determined by qPCR. After PNS and ginsenoside Re treatment for 48 h, total RNA was extracted from 30 larvae using TRIzol reagent (Invitrogen). Reverse-transcription reactions were performed using a reverse transcriptase kit (Takara, Dalian, China). Quantitative real-time PCR with SYBR green system (Takara, Dalian, China) were conducted with β-actin mRNA as reference gene. The primers are listed in Additional File Table S14.

**Results**

Concentration Effect Curves for PNS in Zebrafish Embryos

PNS at 250, 500 and 1000 μg/mL had no effect on WT zebrafish, compared with the control group, while PNS at 2000 μg/mL showed abnormal phenotype. Likewise, ginsenoside Re at 50, 100 and 200 μg/mL had no adverse effects. Therefore, the experimental concentrations of PNS were set as 1000, 500 and 250 μg/mL, and the concentrations of ginsenoside Re were set as 200, 100 and 50 μg/mL (Additional File Table S13). In the case of high-sugar and high-fat model, PNS administered concentrations were set as 1000, 500 and 250 μg/mL as PNS at 2000 μg/mL induced 83% death rate, and the concentrations of ginsenoside Re were set as 25, 12.5 and 6.25 μg/mL, since concentrations higher than 25 μg/mL induced abnormal phenotypes.

Quantitative Analysis of PNS major components

To further validate the chemical variations of PNS, 9 major compounds of PNS, including notoginsenoside R1, ginsenoside Rb1, ginsenoside Rb2, ginsenoside Rd, ginsenoside Re, ginsenoside Rg1, ginsenoside Rg2, ginsenoside Rg3 and ginsenoside Rh1 were quantitatively determined with PRM mode. The results are displayed in Additional File Figure S9. The quantitative product ion and collision energy for each analyte was optimized and data was processed using Skyline software [1] and XcaliburTM 4.1 software (ThermoFisher, San Jose, CA, USA, 2019). The detailed result was listed in Additional File Table S9.

ROS production in zebrafish

Based on morphological assessments of zebrafish embryos, PNS and ginsenoside Re were administered to measure ROS production using fluorescence intensity of CM-H_2_DCFDA probe. PNS and ginsenoside Re treatments both resulted in the decrease of ROS production in WT zebrafish and high-sugar model zebrafish. ROS levels relative to control in WT were 64.0%, 65.1% and 66.9%, respectively, in zebrafish treated with PNS concentrations 250, 500 and 1000 μg/mL. For zebrafish treated with ginsenoside Re concentrations 50, 100 and 200 μg/mL, the ROS levels relative to control were 64.8%, 64.2% and 69.3%, respectively (Figure 8B). Furthermore, ROS levels relative to model control in high-sugar model were 60.0%, 65.3% and 44.5%, respectively, in high-sugar zebrafish treated with PNS concentrations 250, 500 and 1000 μg/mL. For zebrafish treated with ginsenoside Re concentrations 6.25, 12.5 and 25 μg/mL, the ROS levels relative to model control were 71.2%, 72.2% and 75.1%, respectively (Figure 8D).

**References**

1. Adams KJ, Pratt B, Bose N, Dubois LG, St. John-Williams L, Perrott KM, et al. Skyline for small molecules: a unifying software package for quantitative metabolomics. 2020;19(4):1447-58.
